# Trace Metal Bioaccumulation in Feral Pigeons (*Columba livia* f. *domestica*) and Rooks (*Corvus frugilegus*) Residing in the Urban Environment of Iasi City, Romania

**DOI:** 10.3390/toxics12080593

**Published:** 2024-08-16

**Authors:** Diana Iacob, Emanuela Paduraru, Vicentiu-Robert Gabor, Carmen Gache, Iuliana Gabriela Breaban, Silviu Gurlui, Gabriel Plavan, Roxana Jijie, Mircea Nicoara

**Affiliations:** 1 Doctoral School of Geosciences, Faculty of Geography and Geology, Alexandru Ioan Cuza University of Iasi, No. 20A Carol I Avenue, 700505 Iasi, Romania; dianaelenaiacob84@gmail.com (D.I.); emanuelapaduraru19@yahoo.com (E.P.); iulianab2001@yahoo.com (I.G.B.); 2 Department of Geography, Faculty of Geography and Geology, Alexandru Ioan Cuza University of Iasi, No. 20A Carol I Avenue, 700505 Iasi, Romania; gaborvicentiu@gmail.com; 3 Department of Biology, Faculty of Biology, Alexandru Ioan Cuza University of Iasi, No. 20A Carol I Avenue, 700505 Iasi, Romania; cgache@uaic.ro (C.G.); gabriel.plavan@uaic.ro (G.P.); 4 Faculty of Physics, Alexandru Ioan Cuza University of Iasi, No. 11 Carol I Avenue, 700506 Iasi, Romania; sgurlui@uaic.ro; 5 Research Center on Advanced Materials and Technologies (RAMTECH), Department of Exact and Natural Sciences, Institute of Interdisciplinary Research, Alexandru Ioan Cuza University of Iasi, No. 11 Carol I Avenue, 700506 Iasi, Romania; roxana.jijie@uaic.ro

**Keywords:** trace metals, birds, pigeons, rooks, air pollution, urban pollution

## Abstract

Nowadays**,** trace metal contamination within urban atmospheres is a significant and concerning global issue. In the present study, two synanthropic bird species, namely, the feral pigeon (*Columba livia* f. *domestica*) and the rook (*Corvus frugilegus*), were employed as bioindicators to assess the atmospheric trace metal pollution in Iasi City, Romania. The concentrations of Ni, Pb, Cd, Co, Cr, and Cu were determined through high-resolution continuum source graphite furnace atomic absorption spectrometry (HR-CS GF-AAS) of various tissues, including the liver, kidney, lung, heart, muscle, and bone, of feral pigeons and rooks collected in Iasi City. The order of trace metal concentrations in the tissues of feral pigeons and rooks in Iasi City was similar: Cu > Pb > Ni > Cd > Cr > Co. However, trace element values in most tissues were higher in the rook samples than in feral pigeon ones, except for Co, which had elevated levels in feral pigeon renal and cardiac tissues, and Cu, which registered the highest concentrations in feral pigeon liver and kidney tissues. While not statistically significant, Pb concentration values in the PM_10_ fraction of atmospheric particles positively correlated with Pb concentrations in rook kidney samples (*p* = 0.05). The concentration levels of Cd, Pb, and Ni in the PM_10_ fraction of air particles showed a positive correlation with Cd levels in the samples of pigeon heart and rook liver, kidney, and heart, Pb levels in the samples of pigeon kidney, heart, and muscle and rook liver and bone, and Ni levels in the samples of pigeon liver, kidney, and bone and rook liver, muscle, and bone, respectively.

## 1. Introduction

Contamination with toxic elements is a global issue [1,2], particularly trace metal pollution [1], which is undoubtedly one of the most severe problems with environmental, economic, and social dimensions that can be deeply alarming [1,3]. The ecosystem’s stability is significantly threatened by the presence of xenobiotics [1] such as pesticides and trace metals, particularly Cu, Fe, Mn, Zn, Cd, Pb, Cr, and Hg [4,5]. Various trace metals, such as Cd, Pb, and Hg, have no biological role in organisms and become toxic even after chronic exposure to comparatively low concentrations. However, other trace metals are essential elements, namely, Cu, Zn, Fe, Mn, Co, and Ni, and can only reach toxicity at higher exposure levels [6].

Since the early 1960s, biomonitoring has been a critical tool in tracking environmental pollution levels by monitoring sentinel organisms and their ecosystems [7,8]. Biological species employed as bioindicators [7] play a critical role in identifying conclusive evidence of pollution, especially trace metal pollution, which poses a significant environmental threat [1]. Birds have become a preferred option for bioindicators as they are more sensitive to environmental pollution than other vertebrates [3]. They are especially effective indicators of environmental trace metal contamination, focusing on geographic, historical, and global patterns [9]. Due to their high trophic positions [3,10], birds exhibit various dietary preferences [3], inhabit multiple trophic levels within ecosystems [9], and are valuable for environmental monitoring [10]. In addition, birds tend to inhabit certain regions and can easily be obtained for pollution studies [11]. Given their longevity, diverse diets, and ability to measure ecosystem health, birds are essential bioindicators [7]. Birds’ foraging habits significantly impact the accumulation of trace metals in their bodies. Scavengers and raptors acquire higher amounts of trace metals than granivores, which accumulate them at lower levels [12]. Various factors, such as diet, age, gender, and location, also play a role in metal accumulation [13]. The concentration of trace metals in birds’ bodies can indicate the pollution of their habitat [3], with buildup influenced by various factors, including food sources, the exposure duration in the habitat, and physiological issues [10]. Birds are susceptible to accumulating trace metals due to contaminated food, water, and air sources [5,13] resulting from human activities [13]. These metals can be retained, accumulated, or eliminated within the avian body [5], with elimination occurring through various tissues such as the feathers, liver, and kidney [13]. Birds rely on processes such as excretion or storage in glands like the uropygial gland, salt gland, and feathers for metal elimination. Furthermore, female avian species can eliminate metals through their eggs [5]. Notably, birds can eliminate substantial amounts of ingested Pb and Cd through excrement [14]. Prolonged exposure to trace metals can harm avian biological processes, including disruptions in pairing behavior, reproductive success, alterations in feather development rates and immune responses, and changes in various other biological characteristics [15].

Selecting a bird species as a sentinel species requires careful consideration, and choosing commonly found species with widespread distribution is crucial to avoid undue stress on endangered populations. Additionally, utilizing species adapted to diverse habitats allows for comparative analysis across varying environments [3]. Synanthropic organisms, which live near humans in urban environments, may serve as ideal biological models to assess the significance of trace metals in urban settings [15,16]. Among globally synanthropic avian species, feral pigeons (*Columba livia* f. *domestica* Gmelin, 1789) are significant bioindicators due to their ecological and toxicological significance in natural and human-altered environments [17]. Thriving in urban settings, feral pigeon populations are widespread globally. They are distinctive in their biological and ecological characteristics, including their restricted mobility throughout various seasons, elevated metabolic and respiratory rates, and consumption of seeds and feed subject to environmental particle deposition [4,5,18].

Furthermore, they predominantly inhabit urban environments, displaying strong fidelity to their birth sites and remaining within a limited area (<2 km) throughout their lives [16,17]. Studies indicate that these birds possess adaptive capabilities, enabling them to adjust their feeding, nesting, and behavioral patterns to suit anthropogenic landscapes [5,19]. Their adaptability aligns with their exposure to trace metal pollutants, mirroring that of humans, thus substantiating their widespread application in urban pollution surveillance [9,20]. Variation in metal accumulation among feral pigeons across locales underscores the relationship between atmospheric metal concentrations, traffic density, and habitat characteristics [18]. Contemporary assessments of trace element concentrations within European synanthropic organisms are lacking. While investigations into trace metal levels among feral pigeon populations date back several decades in various European nations such as the Netherlands [21], United Kingdom [22], Spain [23,24,25], Croatia [26], Slovakia [20,27], Kosovo [2,16,28,29], and Germany [30], recent inquiries have extended to global contexts, encompassing regions such as Bangladesh [9], Chile [31], China [32,33,34], India [35], Japan [10], Korea [8,18,36,37,38,39,40], Peru [41], Mexico [42], Morocco [43,44], Iraq [1], and Saudi Arabia [11,45]. The waning interest in trace metal research within European studies may stem from the prevailing belief that decreased emissions have led to negligible environmental concentrations and consequently diminished ecological significance. However, this presumption warrants scrutiny as these pollutants persist in the environment, exhibit mobility, and maintain bioavailability. Despite declining emissions, anthropogenic activities continue to release them [15].

Corvid birds, with their global presence and adaptability to human settlements, play a crucial role in our efforts to comprehend the impact of urbanization on wildlife. Furthermore, the Corvidae family is a fascinating group of mid to large-sized passerines. They showcase a remarkable range of adaptability, thriving in diverse urban environments, from the outskirts of suburbs to the bustling cores of cities. Their ability to select and thrive in such habitats has led to the characterization of corvid species as urban adapters and exploiters [46]. The rook (*Corvus frugilegus* Linnaeus, 1758), a highly social bird species, is often found in flocks, colonies, and communal roosts [47] distributed across Europe and central Asia [48]. Environmentally sensitive species like rooks are frequently employed as bioindicators to assess pollution levels within ecosystems. This is primarily due to their susceptibility to environmental changes and capacity to accumulate contaminants from their surroundings [14].

While earlier studies have offered some understanding of trace metal concentrations in omnivorous birds, including corvids, there are still significant gaps. For instance, Horai et al. [10] examined Zn and Cu concentrations in the liver tissues of the jungle crow (*Corvus macrorhynchos* Wagler, 1827), offering valuable comparative data on corvids in Japan. Similarly, in Poland, researchers focused on trace metal accumulation (Cu, Zn, and Pb) in the liver tissues of omnivorous species such as *Corvus frugilegus* [49]. A recent study in Italy utilized hooded crows (*Corvus cornix* Linnaeus, 1758) as bioindicators to detect environmental contaminations caused by toxic trace metals [50]. These studies, while informative, highlight the need for further investigation and a more comprehensive understanding of the topic.

Although recent studies have examined various trace elements’ accumulation in tissues of avian species, few papers have discussed the relationship between atmospheric trace metal pollution and avian trace metal concentrations in urban environments. In this context, our study aimed to assess trace metal concentrations, encompassing a spectrum of elements such as Cd, Co, Cr, Cu, Ni, and Pb, and their spatial distribution within avian tissues to establish the efficacy of two common avian species as sentinel species for atmospheric trace metal pollution in the urban landscape of Iasi City. The study sought to provide a comprehensive understanding of the presence and distribution of these metals and examine if the trace metal concentration levels detected in the birds’ lungs were due to inhalation of atmospheric particles contaminated with trace metals. To the best of our knowledge, there are no previous comprehensive reports on the presence and distribution of all these trace metals in the tissues of feral pigeons (*Columba livia* f. *domestica*) and rooks (*Corvus frugilegus*) and the correlation with atmospheric trace metal pollution in Iasi City or elsewhere.

## 2. Materials and Methods

### 2.1. Study Area

Iasi is a bustling urban agglomeration in the north-eastern region of Romania, with a population exceeding 400,000 people. Multiple residential areas and two significant industrial zones make up the built-up areas in this location. Iasi is a valley city because of its location between hills, which can trap pollutants and affect air quality. Thus, the city and its inhabitants experience air pollution because of high traffic and industrial activity levels [51].

**Figure 1:**
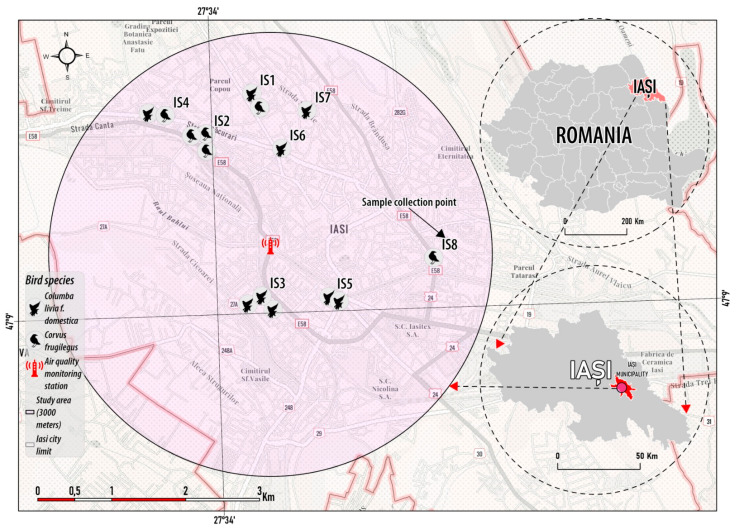
Map of the study area with locations of the sampling sites (IS1–IS8) and the traffic air monitoring station in Iasi City.

Samples were collected from eight sampling sites in Iasi City (labeled as IS1 to IS8 and illustrated in Figure 1), concentrated mainly in the city center and near industrial areas, defined by a characteristic urban setting, with residential and multipurpose buildings, roads with medium to high traffic intensity, and also patches of ornamental and native plant species that provide shelter and food sources to bird species common in these areas.

### 2.2. Sample Collection and Processing

For this study, bird carcasses were randomly collected in eight locations within Iasi City (Table S1 in Supplementary Materials). Nine adult specimens of feral pigeon (*Columba livia* f. *domestica*) and six adult specimens of rook (*Corvus frugilegus*) were collected between November 2019 and November 2021. The bird samples were transported to the laboratory in metal-free polyethylene zip bags on ice and stored in a freezer at −21 °C until trace metal analysis. After thawing, the bird carcasses were weighed and measured, and then necropsied. Most of the specimens had signs of tissue discoloration and internal hemorrhaging; their deaths probably came after a violent impact with hard surfaces. During the necropsy process, various morphological indices (outlined in Table S1 in Supplementary Materials) were meticulously recorded for the subjects sampled in this study. Recorded weight, wing, and tail length indicated that the sampled individuals exhibited normal developmental parameters. Furthermore, all specimens were adults (unfortunately, we did not retrieve any juvenile individuals), with females representing 26.66% of the individuals and males comprising 73.33%. Due to the unequal distribution of age and sex ratios, it was considered inappropriate to include these variables in the analysis of factors influencing trace element bioaccumulation. While the relevance of gender and age is recognized, their inclusion would have needed a more intricate sample structure and a significantly more detailed analysis, which was not feasible within the scope of this study. Although body size and weight, which often differ between genders, are acknowledged to impact trace element bioaccumulation, the focus of this study’s analysis was to provide an overview of bioaccumulation within the studied populations.

The sample preparation procedures followed the steps detailed in the scientific literature [9,52]. However, the amounts of substances used were adjusted to conform with the type of samples and equipment manufacturers’ recommendations. While performing the necropsy procedure on the birds, six samples from each specimen—organs (heart, liver, lung, and kidney), muscle (pectoral muscle), and bone (sternum)—were isolated for chemical analysis. The tissue and bone samples (two replicates for each sample type) were sectioned, cleaned of any debris, rinsed with ultrapure water (18.2 MΩ·cm^−2^, equipment LaboStar^™^ UV 4 Siemens Water Technologies, Barsbüttel, Germany), and air-dried at room temperature (22 °C). For metal analysis, each sample fragment sectioned from the whole organ, muscle, and bone samples was weighed (to approximately 0.5–1 g). Afterward, the previously weighted sample fragments were transferred to decontaminated pressure vessels made of TFMTM–PTFE (DAP-60K type). Then, 4 mL of nitric acid Suprapur^®^ of 65% concentration (certified Merck, Darmstadt, Germany) and 2 mL of hydrogen peroxide EMSURE^®^ ISO of 30% concentration (certified Merck, Darmstadt, Germany) were added. Following the passing of a 30 min reaction time for compounds, the Teflon vials were inserted into a microwave digestion system (SpeedWave MWS-2 Berghof Laborprodukte GmbH, Eningen, Germany) with a 3-step acid digestion program for organic matrix samples. Subsequently, the liquid samples resulting from the microwave digestion process were transferred into 15 mL transparent polypropylene tubes resistant to the corrosive action of acids, and after cooling, they were brought to the mark with ultrapure water (18.2 MΩ·cm^−2^, equipment LaboStar^™^ UV 4 Siemens Water Technologies, Barsbüttel, Germany) in 50 mL clear polypropylene tubes and kept in the laboratory until the spectrometer elemental analysis.

For comparison purposes, the data regarding the air metal concentrations in Iasi City were obtained from the National Environment Protection Agency in Romania [53]. The air samples from Iasi City were collected daily from 2016 to 2020 at an individual collection point within a 3 km radius of the sampling sites for pigeons and rooks and at a height of 40 m from the ground. A traffic air monitoring station collected particulate matter of less than 10 microns (PM_10_) from the air. Furthermore, in a laboratory setting, the researchers from the National Environment Protection Agency in Romania assessed the concentrations of Cd, Pb, and Ni in PM_10_ fractions of atmospheric particles sampled daily using a standard measurement method and atomic absorption spectrometry [53]. It is noteworthy that the atmospheric particles in Iasi City were sampled during a period that coincided with the presence of pigeons and rooks in the area; specifically, the collected bird specimens were all adults approximately 3 to 5 years old prior to their deaths before the sampling dates, indicating that they hatched between 2016 and 2019.

### 2.3. Trace Metal Analysis

In this study, an analytical process based on high-resolution continuum source graphite furnace atomic absorption spectrometry (HR-CS GF-AAS) was employed to evaluate the concentration of metal elements in the various tissue samples of birds, including organs, muscles, and bones. The concentrations of six metals (Cd, Co, Cr, Cu, Ni, and Pb) were determined using a high-resolution continuum source atomic absorption spectrometer equipped with a graphite furnace (equipment ContrAA 600-Analytik Jena, Jena, Germany) and Aspect CS 1.5.6.0 software (Analytik Jena AG, Jena, Germany). The wavelengths used for detecting the signals of Cd, Co, Cr, Cu, Ni, and Pb were 228.8018 nm, 240.7254 nm, 357.8687 nm, 324.754 nm, 232.003 nm, and 217.0005 nm, respectively. To calibrate the equipment for the analyzed metals, a reference standard solution, standard ICP multielement IV (Certipur^®^, 1000 mg·L^−1^, Merck, Darmstadt, Germany), was diluted to 5 µg·L^−1^ for detecting Cd, Co, Cr, Cu, and Pb signals and 20 µg·L^−1^ for detecting Ni signals. Following this, a calibration curve was established using six concentrations, including a blank with ultrapure water and five others that were diluted to specific concentrations (ranging from 0 to 5 for the 5 µg·L^−1^ standard and from 0 to 20 for the 20 µg·L^−1^ standard) by the spectrometer. Linear calibration curves were established for each set of 20 liquid samples per analyzed metal to verify the accuracy of the working method. The coefficient R of the calibration curve did not decrease below 0.995 for any of the elements analyzed in the spectrometer. Moreover, blank and spiked samples were included in each set of experiments to assess the purity of the chemicals. Furthermore, a certified reference material, dry mussel tissue (ERM-CE278k), from the Institute for Reference Materials and Measurements (IRMM) of the European Commission’s Joint Research Centre (JRC) was used to validate and assess the quality of the methodology employed. The reference material was certified for the mass fractions of 13 elements (As, Cd, Cr, Cu, Fe, Hg, Mn, Ni, Pb, Rb, Se, Sr, and Zn). Furthermore, the reference material was processed according to a previously reported protocol [52]. Subsequently, ten samples were prepared from this material, with each sample precisely weighed to attain a mass of 0.3 g. These samples were processed with 4 mL of nitric acid Suprapur^®^ of 65% concentration (certified Merck, Darmstadt, Germany) and 2 mL of hydrogen peroxide EMSURE^®^ ISO of 30% concentration (certified Merck, Darmstadt, Germany) in a microwave digestion system (SpeedWave MWS-2 Berghof Laborprodukte GmbH, Eningen, Germany). The digestion program utilized was similar to the 3-step acid digestion program for organic matrix samples employed for the tissue samples’ analysis, as detailed by Plavan et al. (2017) [54]. After the microwave digestion, the liquid samples were transferred and diluted with ultrapure water in decontaminated 50 mL clear polypropylene tubes. The recovery rates observed in Cd, Cr, Cu, Ni, and Pb ranged from 97% to 105%, indicating satisfactory results. The method detection limits (LOD) were determined as Cd: 0.15; Co: 0.1256; Cr: 0.1178; Cu: 0.098; Ni: 0.382; and Pb: 0.2716, expressed as µg·L^−1^. The spectrometer took two readings for each liquid sample, and duplicates of each tissue sample of organs, muscle, and bone were prepared. The average of those readings was used to obtain the sample analysis result. The absorbance signal of the elements was checked and compared to the values in the calibration curve during each reading, and no errors or interferences were observed in the signal readings, thus ensuring precise and accurate results corresponding to the concentrations of the analyzed elements in the samples. The data obtained after spectrometer measurements were expressed in µg·g^−1^ wet weight (w.w.).

### 2.4. Data Analysis

For the statistical analyses, a widely recognized statistical analysis software in academic research, GraphPad Prism, version 8.4.2.679 for Windows (GraphPad Prism Software, San Diego, CA, USA), was applied. The graphs’ representations used the previously mentioned software. In this study, a Shapiro–Wilk test was employed to assess the normality of the datasets on the trace metal concentrations in the organ, muscle, and bone samples from pigeons and rooks in Iasi City, Romania. A Kruskal–Wallis test was applied to examine the statistical significance of the two bird species, their tissues, and their interactions with the trace metal concentration levels detected. For determining the strength of a relationship and the significance levels applicable for trace metals registered by the National Environment Protection Agency in Romania in the PM_10_ fraction of air samples collected in Iasi City and the trace metal concentration levels measured in tissues of the two common bird species in Iasi City, a Spearman’s rank correlation coefficient was used. A significance level of *p* < 0.05 was established for all analyses, a commonly accepted statistical significance threshold in the field. Where concentration values fell below the detection limits, they were handled according to the standard practices of the field, which involved dividing the detection limit by the square root of two, ensuring the validity and reliability of this research.

The literature concentrations reported on a dry weight (d.w.) basis were adjusted to w.w. using the moisture levels provided by Cui et al. [34] for the liver (68%), kidney (73%), and lung (74%). Additionally, the moisture content documented by Ohlendorf and Heinz [55] at 74% was utilized for muscle. Franson and Pain [56] stated that 1 µg·g^−1^ w.w. approximately equals 1.2 µg·g^−1^ d.w. in bone samples of mallards (*Anas platyrhynchos* Linnaeus, 1758).

## 3. Results

### 3.1. Body Burden of Trace Metals in Pigeons and Rooks in Iasi City

The trace metal concentration values measured in the study indicated a relatively low level of variability among the investigated trace elements, as described in Table 1 and Table 2. These findings offer valuable insights into the distribution of trace metals in pigeons and rooks, underscoring the necessity for further exploration in this domain.

Table 1 showcases the distribution of trace elements in pigeons from Iasi City. Notably, the concentration of trace metals in pigeon tissues followed a distinct pattern, with Cu being the most prevalent, followed by Pb, Ni, Cd, Cr, and Co. Furthermore, the accumulation of trace metals in the tissues of pigeons exhibited a significant grading order of bone > kidney > liver > heart > muscle > lung. Cu had the highest concentrations in almost all tissues except for bone, where Pb and Ni were most abundant. A significant buildup of Cu in the liver and a prevalence of Cd and Co in the kidney was observed. In addition, bone and kidney had the highest average amounts of Cr.

The assessment of trace element concentrations in the bodies of rooks in Iasi City is represented in Table 2. The order of trace metal concentration in the tissues of rooks in Iasi City was similar to that of pigeons: Cu > Pb > Ni > Cd > Cr > Co. The accumulation of trace metals in rooks followed a grading order of bone > heart > liver > muscle > kidney > lung. Cu was the predominant trace element in almost all examined tissues except bone, where Pb and Ni were the most abundant trace metals. Moreover, Cu had a significant buildup in the heart and pectoral muscles. Cd was notably more substantial in the kidney and liver, while Co exhibited elevated levels in bone samples. Both muscle and bone had the highest average amounts of Cr.

Trace element analysis revealed that the trace metal concentration values measured in the tissues of the two bird species in Iasi City did not follow a normal distribution. As a result, a nonparametric test was used for further investigation. Significant differences in the accumulation of Cd, Cr, Cu, Ni, and Pb were discovered in the tissues of the bird species (*p* < 0.0001). Additionally, statistically significant associations were observed between bird species, tissues, and metals, particularly for Cd, Cu, Ni, and Pb (*p* < 0.0001). However, it is important to interpret these results cautiously due to the small and uneven sample size of the bird specimens collected in Iasi City for this study.

### 3.2. Comparison between the Body Burden of Trace Metals in Pigeons and Rooks in Iasi City

**Figure 2:**
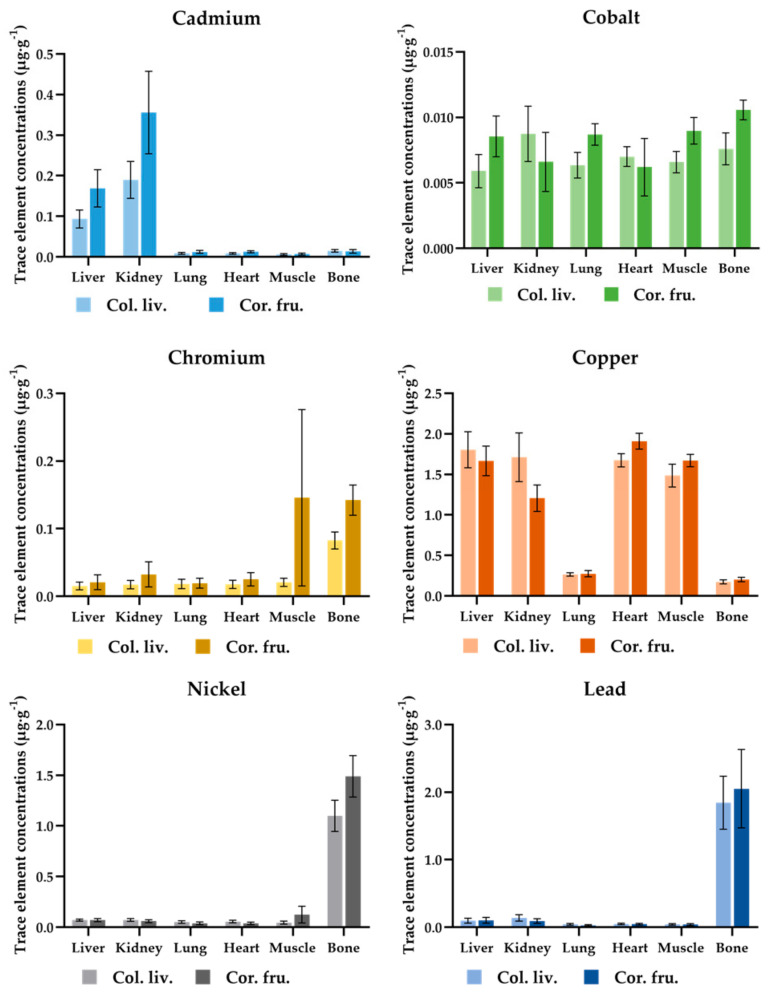
Comparison of trace metal concentrations (µg·g^−1^) in the different sample types from feral pigeons (*Columba livia* f. *domestica*) and rooks (*Corvus frugilegus*) in Iasi City (Romania).

In the comparative evaluation illustrated in Figure 2, the trace metal concentrations in the tissues of pigeons (*Columba livia* f. *domestica*) and rooks (*Corvus frugilegus*) in Iasi City were examined.

The rooks exhibited the highest values of Cd concentration across all the body tissues studied, while the lowest Cd concentration was registered in pigeons. Notably, significant variations in Cd concentrations between the two bird species were evident in the liver and kidney, while no discernible differences were observed in the other analyzed tissues. Furthermore, trace metal accumulation (Cd) in the kidney was notably higher in rooks than in pigeons. The concentration levels of Co in the examined samples varied across both avian species. Most Co concentrations detected in the pigeon samples were lower than those observed in rooks, except for higher levels found in pigeon renal and cardiac tissues compared to rooks. Moreover, substantial disparities were revealed in the Cr concentration levels between pigeons and rooks, with pigeons demonstrating lower levels of Cr concentration, particularly in muscle and bone samples. In the comparative analysis of Cu concentration levels in the liver and kidney samples obtained from pigeons and rooks, the results indicated lower Cu levels in rooks. Conversely, Cu concentration values observed in heart, muscle, and bone samples exhibited slightly elevated levels in rooks. Pigeon and rook bone samples exhibited the highest Ni and Pb concentration levels, with rook samples demonstrating higher concentration values.

### 3.3. Comparison of Trace Metal Concentrations in the PM_10_ Fraction of Air Particles with the Body Burden of Trace Metals in Pigeons and Rooks in Iasi City

The analysis of the annual mean concentrations of trace metals (Cd, Pb, and Ni) in the atmospheric particles (PM_10_) in Iasi City (Table 3) revealed that they were within the thresholds established in legislation (5 µg·m^−3^, 20 ng·m^−3^, and 0.5 ng·m^−3^) [53].

A comparison was performed between the air metal concentrations in Iasi City, recorded by the National Environment Protection Agency in Romania [53], and the concentrations measured in the organs, muscle, bone, and specifically in the lung tissue of pigeons and rooks. No statistically significant correlations were found between heavy metal concentrations in tissues and the PM_10_ fraction of air particles. However, the most pronounced positive correlation was observed between the Pb levels of concentration in the PM_10_ fraction of air particles and the Pb concentrations in rook kidney samples (*p* = 0.05).

**Figure 3:**
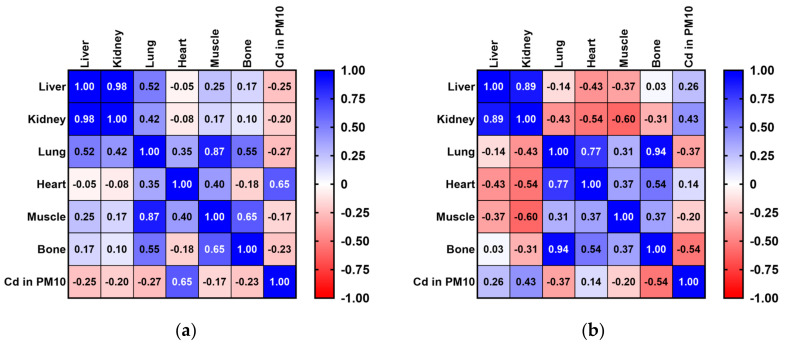
Correlation matrix heatmap (Spearman’s rank correlation coefficient—rs was used to compute the relevance and redundancy of the features) showing the relationship between Cd concentrations in the PM_10_ fraction of air particles in Iasi City and (**a**) Cd concentrations in the organ, muscle, and bone samples from pigeons in Iasi City and (**b**) Cd concentrations in the organ, muscle, and bone samples from rooks in Iasi City. The colors correspond to the levels of correlation: with 1 indicating a positive correlation (dark blue) and −1 indicating a negative correlation (dark red).

The measured amounts of Cd in lung samples from pigeons and rooks exhibited low negative correlations with the Cd values of the PM_10_ fraction in atmospheric particles. Although not statistically significant, low positive correlations were observed among the Cd concentration levels in the PM_10_ fraction of air particles and Cd concentrations in pigeon heart samples (Figure 3a). In contrast, Cd concentration values for rook liver, kidney, and heart samples registered weak positive correlations with air Cd levels (Figure 3b).

**Figure 4:**
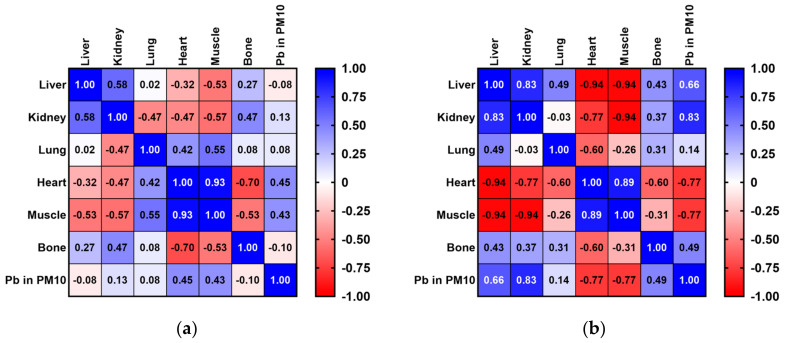
Correlation matrix heatmap (Spearman’s rank correlation coefficient—rs was used to compute the relevance and redundancy of the features) showing the relationship between Pb concentrations in the PM_10_ fraction of air particles in Iasi City and (**a**) Pb concentrations in the organ, muscle, and bone samples from pigeons in Iasi City and (**b**) Pb concentrations in the organ, muscle, and bone samples from rooks in Iasi City. The colors correspond to the levels of correlation: with 1 indicating a positive correlation (dark blue) and −1 indicating a negative correlation (dark red).

The Spearman’s rank correlation coefficient revealed similar inferior positive values for Pb concentration values in the lung samples of pigeons and rooks compared with the Pb concentration levels in the PM_10_ fraction of air particles. Furthermore, slightly higher positive correlations were observed between the Pb concentration values of pigeon kidney, heart, and muscle samples and Pb air levels in the PM_10_ fraction (Figure 4a). However, a weaker positive correlation was revealed in the Pb concentration values of rook liver and bone samples with Pb concentration levels in the PM_10_ fraction of air particles (Figure 4b).

**Figure 5:**
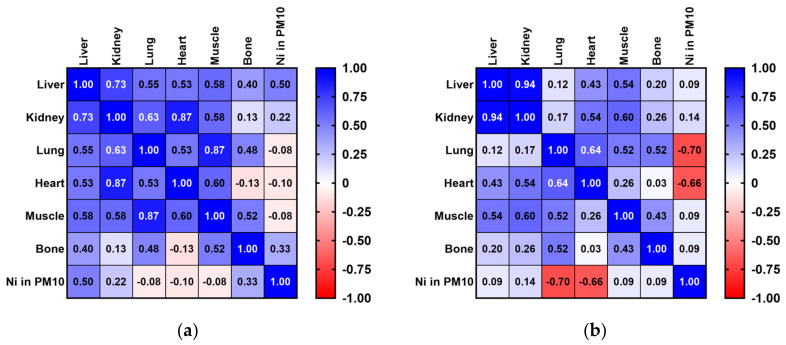
Correlation matrix heatmap (Spearman’s rank correlation coefficient—rs was used to compute the relevance and redundancy of the features) showing the relationship between Ni concentrations in the PM_10_ fraction of air particles in Iasi City and (**a**) Ni concentrations in the organ, muscle, and bone samples from pigeons in Iasi City and (**b**) Ni concentrations in the organ, muscle, and bone samples from rooks in Iasi City. The colors correspond to the levels of correlation: with 1 indicating a positive correlation (dark blue) and −1 indicating a negative correlation (dark red).

Ni concentrations in pigeon lung samples registered a low negative correlation with Ni concentrations in the PM_10_ fraction of air particles (Figure 5a). In contrast, Ni concentrations in rook lung samples measured a higher negative correlation with the Ni air values. However, the results of the Ni concentrations detected in pigeon liver, kidney, and bone samples had weak positive correlations with Ni concentration values in the PM_10_ fraction of air particles, although these correlations were not found to be significant. Furthermore, the Ni amounts measured in the rook liver, kidney, muscle, and bone samples correlated positively with Ni concentration values in the PM_10_ fraction of air particles (Figure 5b).

## 4. Discussion

Previously, the literature documented trace metal concentrations in sediments and bioindicator organisms in Iasi City, Romania [57,58]. Nonetheless, recent Romanian studies have presented reports on trace metal concentrations in noninvasive samples such as feathers and eggshells from various bird species [59,60,61], as well as Pb concentrations in tissues [62,63]. However, there is no reported data on trace metal pollution in pigeons and rooks in Iasi City.

The focus of this study was the evaluation of two common bird species as sentinel species for environmental pollution with trace metals in the urban environment of Iasi City, with emphasis on the possible route of exposure being the inhalation of toxic substances. Furthermore, chronic exposure, even at low doses, to urban atmospheric pollution caused by various factors, including traffic exhaust, can cause long-term effects on living organisms. With atmospheric exposure to trace metals, a correlation between the levels of trace metal concentrations from the PM_10_ fraction of air particles and the trace metal concentration values in the lung samples of exposed organisms would have been expected. Although the results of this research did not reveal any statistically significant correlation among these variables, low positive correlations were observed between Pb concentration levels in the PM_10_ fraction of air particles and Pb concentration values in the lung samples of pigeons and rooks. However, Ni concentration levels in pigeon lung samples exhibited a weak negative correlation with Ni concentration levels in the PM_10_ fraction of air particles, while Ni concentration levels in rook lung samples registered a higher negative correlation with the Ni air values. Cd concentration levels in the lung samples from pigeons and rooks exhibited a lower negative correlation with the amount of Cd detected in the PM_10_ fraction of atmospheric particles. Although the annual mean values for trace element concentrations in the PM_10_ fraction of air particles collected between 2016 and 2020 in Iasi City showed a descending trend (Table 3), and lower concentration values for trace metals in the lung tissue of pigeons and rooks residing in this area were measured, it cannot be stated with certainty that their exposure was not due to the inhalation of these toxic substances because researchers agree that constant exposure to small doses of these pollutants may have chronic effects on living organisms. Moreover, insufficient evidence exists regarding the specific interactions between inhalation and dietary exposure pathways. The limited and disproportionate sample size of the specimens collected from the two bird species in this study further adds complexity to the interpretation of the findings.

Scientists have explored the correlations between the concentrations of trace elements accumulated in birds’ tissues and their trophic levels [64]. Thus, the differences observed in the bioaccumulation of heavy metals in the tissues of the two bird species in Iasi City may also be due to their type of diet. Therefore, the rook (*Corvus frugilegus*), an omnivorous species, accumulated higher concentrations of the analyzed elements in tissues than the domestic pigeon (*Columba livia* f. *domestica*), a granivorous species.

On the other hand, higher concentrations of Cd were measured in liver and kidney samples compared to the lung tissue of pigeons and rooks in Iasi City, thus confirming that as metabolically active tissues, the liver and kidney are more susceptible to accumulating trace metals, making them valuable indicators of chronic exposure [13]. Thus, a liver-to-kidney ratio < 1 of Cd concentration levels, similar to the one registered in this study, points to chronic exposure to lower levels of this contaminant [65], regardless of whether the exposure route was respiratory or ingestion.

Furthermore, during a comprehensive analysis of the literature data on trace metal accumulation in the tissues of pigeons, rooks, and related species in different pollution gradients, it was evaluated whether the measured trace metal concentration values in Iasi City were within the background environmental pollution thresholds.

### 4.1. Comparison of Cd Concentrations with Literature Values

Cd, a nonessential metal [66], poses a significant threat to kidney health, destroying cells and tissues [26]. It acts as a potent nephrotoxin [26,67], often accumulating in the kidneys at higher concentrations than in the liver [67]. However, various studies have reported similar Cd concentrations levels in both organs [67]. Researchers noted that the route of ingestion rather than inhalation influences the accumulation of Cd in soft tissues, such as the kidneys and liver [39]. Alarmingly, even low concentrations of this trace metal can unleash a cascade of toxic effects, including liver and kidney lesions, growth dysfunction, anorexia, and adverse impacts on reproduction and survival [2,50].

Information on the threshold values for Cd concentrations is limited to specific soft tissues. However, these values should be interpreted cautiously due to various factors affecting Cd accumulation, such as differences in sensitivity to Cd among species, age and diet variations, and environmental influences. The scientific community generally agrees that adult birds’ hepatic Cd threshold level may range from 45 to 70 µg·g^−1^ w.w. for Cd-related effects. A concentration of around 65 µg·g^−1^ w.w. in the kidneys would be linked to a 50% likelihood of changes in energy metabolism or structural/functional damage to tissues, including the kidneys, testes, liver, and gut. Bone Cd levels ≥ 1 µg·g^−1^ w.w. have been associated with adverse effects, such as structural damage to the kidneys and testes, intestinal lesions, or the absence of spermatogenesis in adult experimental birds [68].

Based on the comprehensive literature analysis of Cd concentrations in various tissues from feral pigeons and related species (Table S2 in Supplementary Materials), the mean hepatic value for Cd in feral pigeons in Iasi City, 0.094 ± 0.066 µg·g^−1^ w.w., was slightly higher than those found in the livers of pigeons (*Columba livia*) from different geographic locations (Morocco, Peru, and Korea). For instance, in rural areas in Mohammedia, Morocco, the Cd concentration was 0.05 ± 0.02 µg·g^−1^ w.w. [44], in Allal Behraoui, Rabat-Salé, Morocco, it was 0.07 ± 0.03 mg·kg^−1^ w.w. [43], in Lurín, Peru, it was 0.054 ± 0.078 mg·kg^−1^ w.w. [41], and in an urban area in Kwangju, Korea, it was 0.08 ± 0.02 µg·g^−1^ w.w. [39]. Most rural and urban areas in European countries had liver Cd concentrations within the background threshold levels of 0–3 mg·kg^−1^ d.w. [48], except for an airport area in Heathrow, Middlesex, United Kingdom, where it was measured at 9.48 ± 3.15 µg·g^−1^ d.w. [22], surpassing the subclinical toxicity threshold for Cd (>3 mg·kg^−1^ d.w.) [9]. Outside of Europe, in India (8.59 ± 1.53 µg·g^−1^ w.w.) [35] and Chile (11.695 ± 8.38 µg·g^−1^ d.w.) [31], subclinical toxicity levels for Cd were recorded in the livers of pigeons (*Columba livia*). Notably, the highest Cd concentration value in pigeon liver samples from the scientific literature was found in an urban area in Shaqraa Province, Riyadh, Saudi Arabia—10.65 ± 1.42 mg·g^−1^ w.w. [45]—exceeding the Cd poisoning threshold of 40 mg·kg^−1^ d.w. [50] and being ten-times greater than the concentration recorded in Iasi City pigeons.

The value of Iasi City’s feral pigeon Cd concentration in kidneys at 0.190 ± 0.136 µg·g^−1^ w.w. was similar to that of an urban area of Kwangju, Korea—0.18 ± 0.06 µg·g^−1^ w.w. [39]—and three-times higher than another rural area in Duckjuk Island, Korea—0.06 ± 0.03 µg·g^−1^ w.w. [39]. In Europe, kidney Cd concentrations above the threshold for possible environmental Cd exposure (>2.4 mg·kg^−1^ w.w.) [26] were registered in the Netherlands, in two urban areas in Amsterdam with medium and high traffic levels—2.51 ± 2.8 µg·g^−1^ w.w. and 2.73 ± 2.61 µg·g^−1^ w.w. [21]—in Kosovo, in a rural area in Lubizdë—10 ± 11 µg·g^−1^ d.w.—and in an industrial area in Drenas—19.1 ± 3.6 µg·g^−1^ d.w. [28]—and in the United Kingdom, in an urban area from Chelsea, London—12.3 ± 2.05 µg·g^−1^ d.w.—and an airport area from Heathrow, Middlesex—50.7 ± 22.7 µg·g^−1^ d.w. [22]. Pigeons from an urban area in Shaqraa Province, Riyadh, Saudi Arabia, registered the highest Cd concentration in renal tissue—8.55 ± 0.28 mg·g^−1^ w.w. [45]—which surpassed the Cd toxicosis threshold of 100 mg·kg^−1^ d.w. [67].

Results of Cd concentrations in the pulmonary tissue of Iasi City pigeons revealed a mean value of 0.009 ± 0.007 µg·g^−1^ w.w., which was comparable with the mean lung Cd concentration of pigeons in the industrial area of Oulja, Rabat-Salé, Morocco, which was measured at 0.008 ± 0.002 mg·kg^−1^ w.w. [43]. The diversity of Cd lung levels globally is striking, ranging from almost two-times to one-hundred-times higher than the levels observed in Iasi. For instance, a rural area in Duckjuk Island, Korea, showed Cd mean levels of 0.09 ± 0.05 µg·g^−1^ w.w. [18], which was approximately ten-times higher than that observed in the urban environment of Iasi. On the other hand, captive pigeons from a city in Shaqraa Province, Riyadh, Saudi Arabia, had the highest amount of Cd in their lung samples (7.98 ± 0.93 mg·g^−1^ w.w.) [45], a value significantly higher than the average found in Iasi City.

Pigeon cardiac tissue from Iasi City registered a mean value of 0.008 ± 0.006 µg·g^−1^ w.w. that was two-times lower than the amount of Cd in the hearts of pigeons in a medium-traffic urban area in Rabat-Salé, Morocco (0.019 ± 0.008 mg·kg^−1^ w.w.) [43]. However, it was higher than Cd levels from an industrial area in Oulja, Rabat-Salé, Morocco (0.002 ± 0.001 mg·kg^−1^ w.w.) [43]. The highest average values for heart Cd concentrations were found in Saudi Arabia, reaching 7.87 ± 0.87 mg·g^−1^ w.w. [45] in captive pigeons from an urban area in Shaqraa Province, Riyadh.

The investigation into Cd concentrations in the pectoral muscle tissue of Iasi City pigeons uncovered a mean value of 0.006 ± 0.006 µg·g^−1^ w.w. This mean value is comparable to pigeons’ mean muscle Cd concentration in the urban environment of Santa Cruz de Tenerife, Canary Islands, Spain (0.0075 µg·g^−1^ w.w.) [23] and in several urban areas in Bangladesh (0.03 ± 0.006 µg·g^−1^ d.w.) [9]. The variety of Cd muscle levels globally is remarkable, with concentrations ranging from five to fifty-times higher than in Iasi. Surprisingly, the lowest value for muscle Cd concentration was recorded in a Japanese airport area in Tokyo, 0.00874 ± 0.00885 µg·g^−1^ d.w. [10], which was approximately two-times lower than that observed in the urban environment of Iasi. However, captive pigeons from a city in Shaqraa Province, Riyadh, Saudi Arabia, had the highest amount of Cd in their muscles (3.76 ± 0.67 mg·g^−1^ w.w.) [45], a relatively higher level than the one found in Iasi City.

In a study conducted in Chile, researchers observed that pigeons in Arica exhibited the highest average Cd value in their femurs (3.427 ± 2.75 µg·g^−1^ d.w.) [31]. This figure surpasses the bone Cd levels ≥ 1 µg·g^−1^ w.w. that have been associated with adverse effects in adult experimental birds, as noted by Wayland and Scheuhammer [68]. In contrast, the Cd level in pigeons’ sternums from Iasi City was significantly lower at 0.015 ± 0.010 µg·g^−1^ w.w. Furthermore, the mean Cd bone concentration in an industrial area in Seoul was four-times higher (0.06 ± 0.05 µg·g^−1^ w.w.) than in Iasi City [37]. In the same study, researchers reported Cd concentrations of 0.12 ± 0.02 µg·g^−1^ w.w. in bone samples in a high-traffic urban area in Seoul, Korea [37].

Upon review of the globally comparable studies on Cd concentrations in various tissues from rooks and related species (Table S3 in Supplementary Materials), it was determined that Iasi City’s mean hepatic Cd value for rooks, 0.169 ± 0.112 µg·g^−1^ w.w., slightly exceeded the hepatic Cd concentration found in the livers of hooded crows (*Corvus cornix*) in urban areas in Cuneo Plain, Italy, which was recorded at 0.15 ± 0.26 mg·kg^−1^ w.w. [50]. Worldwide hepatic Cd concentration levels in corvids predominantly were within the background threshold of 0–3 mg·kg^−1^ d.w. [48]. However, the mean concentration value for Polish rooks from various urban environments was notably higher, measuring 17.2 mg·kg^−1^ d.w. [67], and classified as a subclinical toxicity case for Cd (>3 mg·kg^−1^ d.w.) [9]. Furthermore, the highest Cd liver levels in corvids were recorded in house crows (*Corvus splendens* Vieillot, 1817) in India, quantified at 8.78 ± 1.87 µg·g^−1^ w.w. [35]. In contrast, the lowest Cd mean liver concentration was observed in jungle crows (*Corvus macrorhynchus*) from an airport in Tokyo, Japan, measuring 0.253 ± 0.421 µg·g^−1^ d.w. [10].

The rooks’ renal Cd concentration in Iasi City, which was measured at 0.356 ± 0.249 µg·g^−1^ w.w., was higher than the levels found in hooded crows (*Corvus cornix*) from urban areas in Cuneo Plain, Italy, which were reported at 0.15 ± 0.26 mg·kg^−1^ w.w. [50]. Furthermore, this concentration was two to three-times higher than the levels of Cd found in the kidneys of jungle crows (*Corvus macrorhynchus*) and carrion crows (*Corvus corone* Linnaeus, 1758) from an airport in Tokyo, Japan, measuring 0.404 ± 0.621 µg·g^−1^ d.w. and 0.599 ± 0.526 µg·g^−1^ d.w., respectively [10]. Notably, significant kidney Cd concentrations above the threshold for potential environmental Cd exposure (>2.4 mg·kg^−1^ w.w.) [26] were observed in the renal tissue of Polish rooks from various urban environments, averaging 17.0 mg·kg^−1^ d.w. [67]. However, the highest renal Cd levels among corvids were reported in house crows (*Corvus splendens*) from India, quantified at 10.52 ± 5.25 µg·g^−1^ w.w. [35].

In this research, the average concentration of Cd in the pulmonary tissue of adult rooks in Iasi City was 0.012 ± 0.008 µg·g^−1^ w.w. This value surpasses the mean lung Cd concentration of jungle crows (*Corvus macrorhynchus*) and was twice as high as that of carrion crows (*Corvus corone*) at an airport in Tokyo, Japan, which measured 0.404 ± 0.621 µg·g^−1^ d.w. and 0.599 ± 0.526 µg·g^−1^ d.w., respectively [10]. Moreover, rook nestlings from various cities in Poland exhibited the highest amount of Cd in their lung, measuring 17.2 mg·kg^−1^ d.w. [67]. This measurement significantly exceeds the average observed in Iasi City.

The mean value of Cd in rook cardiac tissue in Iasi City was 0.013 ± 0.006 µg·g^−1^ w.w., which was significantly lower than the amount of Cd in the hearts of house crows (*Corvus splendens*)—2.03 ± 0.30 µg·g^−1^ d.w.—from the urban environment of Klang, Malaysia [69].

The investigation of Cd concentrations in the pectoral muscle tissue of Iasi City rooks uncovered a mean value of 0.007 ± 0.006 µg·g^−1^ w.w. This amount exceeds the mean muscle Cd concentration of jungle crows (*Corvus macrorhynchus*) and carrion crows (*Corvus corone*) at an airport in Tokyo, Japan, which measured 0.00493 ± 0.00236 µg·g^−1^ d.w. and 0.00554 ± 0.00286 µg·g^−1^ d.w., respectively [10], by a factor of five. However, results in Iasi City were significantly lower than the mean muscle Cd concentration of house crows (*Corvus splendens*) in the urban area of Klang, Malaysia, which was determined as 0.66 ± 0.13 µg·g^−1^ d.w. [69]. While Polish rook nestlings from various cities in Poland exhibited extremely high Cd in muscle tissue at 17.2 mg·kg^−1^ d.w. [67], the highest Cd amount was detected in the muscles of house crows (*Corvus splendens*) in India, quantified at 5.544 ± 3.56 µg·g^−1^ w.w. [35].

A Polish research study uncovered that rook nestlings in urban areas exhibited the highest average Cd value in their femurs (17.2 mg·kg^−1^ d.w.) [67]. This value exceeds the bone Cd levels ≥ 1 µg·g^−1^ w.w., which were associated with adverse effects in adult experimental birds, as Wayland and Scheuhammer [68] reported. On the contrary, the Cd level in adult rooks’ sternums from Iasi City was significantly lower at 0.014 ± 0.011 µg·g^−1^ w.w. Moreover, these results were found to be lower than the bone Cd levels detected in adult and juvenile specimens of house crows (*Corvus splendens*) in the urban area of Klang, Malaysia, where the mean was determined to be 1.54 ± 0.27 µg·g^−1^ d.w. [69].

### 4.2. Comparison of Co Concentrations with Literature Values

The essential element Co [67,70] is crucial for metabolism [70], specifically for young birds’ growth and development [67]. However, excessive concentrations of Co can have adverse effects [70] and disrupt metabolism [67]. As a part of vitamin B12, Co is associated with nitrogen assimilation and synthesizing hemoglobin and muscle protein. It also influences certain enzymes [70]. While Co is readily available in various foods [71], excessive intake can produce toxic effects on organisms [70]. The mechanism of Co toxicity remains unclear, but it may inhibit crucial enzymes, displace divalent cations in metal-activated enzymes, and compete with calcium (Ca)-binding proteins, among other actions [72]. Co^2+^ can also generate reactive oxygen species through Fenton-like reactions, leading to oxidative stress and deoxyribonucleic acid (DNA), protein, and lipid damage [72]. Co primarily accumulates in the thymus gland [73], liver [73,74], kidneys, heart, spleen, stomach [74], marrow, muscles [73], and pancreas [72,73], with its relative content in the skeleton and skeletal muscles increasing over time following Co administration [72].

Renal tissue Co values for feral pigeons in Iasi City were the highest among all analyzed pigeon samples (0.009 ± 0.006 µg·g^−1^ w.w.). However, compared with values from the literature (Table S4 in Supplementary Materials), they were remarkably lower than the mean Co levels in the kidneys of pigeons in urban medium-traffic areas in Pakistan (Lahore, Punjab), registered at 0.56 mg·kg^−1^ d.w. [71]. Furthermore, the highest Co mean concentration (32.9 ± 4.98 mg·g^−1^ w.w.) was measured in captive pigeon kidneys in Saudi Arabian (Shaqraa Province, Riyadh) urban environments [45], significantly higher than in Iasi City. On the other hand, the bone Cu concentration levels in rooks were the most elevated among all examined rook tissues in Iasi City (0.011 ± 0.002 µg·g^−1^ w.w.). Worldwide, the lowest mean Co concentration was registered in the bone samples of rooks in urban and suburban areas in Russia (Rostovskaya oblast, Azov District) at 0.08 µg·g^−1^ w.w. [75]; this mean value was higher than that of Iasi City. Surprisingly, this exact level was significantly lower than the average Co in the femurs of pigeons in rural habitats in Poland, at 4.1 mg·kg^−1^ d.w. [67].

### 4.3. Comparison of Cr Concentrations with Literature Values

In the environment, there are two oxidation states of Cr that need to be considered: highly toxic Cr (VI) and important trace element Cr (III) [49]. Wild birds and mammals primarily encounter Cr through food ingestion. Notably, improved membrane permeability of Cr (VI) compounds generates a significantly more efficient absorption from the gastrointestinal tract than inorganic Cr (III) compounds [76]. The skeletal system serves as the primary deposition site for Cr [77]. However, due to the scarcity of toxicological data on free-living wildlife species, the toxicological significance of “elevated” Cr concentrations remains unclear [76]. Nonetheless, sublethal effects of Cr in birds incorporate testicular damage, anemia, and growth retardation, which are considered life-threatening to the organism [35]. Data on Cr concentrations in avian wildlife species are limited [76]. Eisler (1986) suggested total Cr tissue levels exceeding 4.0 mg·kg^−1^ d.w. could be indicative of Cr contamination, although the consequences of tissue Cr residues remain ambiguous [78]. Inhabitants of environments remote from Cr contamination sources exhibit tissue concentrations ranging from approximately 0.1 to 15 µg·g^−1^ d.w., depending on the species and tissue analyzed. Levels in organisms experiencing Cr pollution can be twice as high [76]. Cr is typically contained in the skeleton of small rodent species at concentrations of 0.9 µg·g^−1^ [77].

With bone tissue being the predominant accumulation site for Cr, the mean Cr value for rooks’ sternums in Iasi City was compared with worldwide levels. Iasi City’s rook Cr sternum values (0.1422 ± 0.0547 µg·g^−1^ w.w.) were below the typical levels stated in the scientific literature and significantly lower than the highest Cr mean concentration measured in the tibia samples of rooks in urban areas in Russia—8.83 ± 5.29 µg·g^−1^ d.w. [79]. Although there were not any references to worldwide bone Cr levels in pigeons, the mean value for Cr in the sternum samples of feral pigeons in Iasi City (0.083 ± 0.038 µg·g^−1^ w.w.) was considerably lower than the typical Cr bone level of 0.9 µg·g^−1^ described by Lebedeva (1997) for small rodent species [77].

### 4.4. Comparison of Cu Concentrations with Literature Values

The environmental release of Cu [80], an essential metal [66], is a result of mining, smelting activities, and agricultural waste disposal [80]. On the other hand, Cu plays a pivotal biological role [9] in growth, development, metabolism, and maintaining protein structure and function within cells [80]. This essential element is crucial for various enzymatic reactions [1,67] and metabolic processes [1] and is particularly vital for the growth and development of young birds [67]. However, while Cu is essential in appropriate concentrations, high levels can lead to toxicity [2,9,45], resulting in metabolic and growth disturbances, particularly in nestlings [67]. Research indicates that Zn can inhibit Cu accumulation in animal tissues, protecting against its toxic effects [45]. Furthermore, the concentration of Cu is metabolically regulated [23], and the liver levels are mostly below the limit of 50 μg·g^−1^ d.w. [9]. However, it fluctuates according to the physiological requirements during molt and breeding [23]. Prolonged exposure to Cu, even in low doses, can lead to chronic intoxication and accumulation in organisms, particularly in the liver and kidneys, which can lead to toxicity and potential cancer [2]. The liver concentration, a reliable marker of Cu exposure in animals, is considered the primary site for Cu accumulation [9]. In addition, Cu concentration in the liver is influenced by diverse aspects such as the chemical form of Cu, diet, and species, which determines the threshold indicating Cu poisoning in birds. Notably, the liver’s Cu concentration has been linked to health issues in Canadian geese, with high concentrations of Cu (56 to 97 mg·kg^−1^ w.w.) leading to upper gastrointestinal necrosis. On the contrary, chickens and turkeys usually exhibit lower levels of Cu concentration in the liver (12.7 and 17 mg·kg^−1^ d.w., respectively), indicating the variation in the impact of Cu across different avian species [26].

The scientific literature values presented for pigeons, rooks, and related species were analyzed to uncover whether Cu concentrations vary across species and distinct environment types. Hepatic tissue was analyzed due to its propensity for Cu accumulation. Moreover, the liver concentrations of feral pigeons and rooks in Iasi City was 1.804 ± 0.663 µg·g^−1^ w.w. and 1.666 ± 0.447 µg·g^−1^ w.w., respectively. The pigeons’ hepatic Cu values measured in this study were slightly higher than those in the livers of pigeons in the industrial areas from Seoul, Korea (1.61 ± 0.80 µg·g^−1^ w.w.) [37]. However, they were significantly lower than the levels recorded in pigeons collected from an industrial area in Drenas, Kosovo (18.48 ± 5.43 µg·mg^−1^ d.w.) [2], which had the highest value and surpassed the limits linked with gastrointestinal impairment (56 to 97 mg·kg^−1^ w.w.) stated by Prevendar Crnić and his research team [26]. Surprisingly, captive pigeons in an urban environment in Al-Nassiriyah, Iraq, revealed the lowest Cu liver mean concentration at 0.164 ± 0.02 µg·g^−1^ d.w. [1]. The adult rooks in Iasi City had hepatic Cu values almost twice as high as those from nestling rook specimens in urban areas in Poland [67]. Furthermore, they were surprisingly lower than the average Cu levels in the livers of house crows (*Corvus splendes*) in India (45.03 ± 10.64 µg·g^−1^ w.w.) [35]. These figures were within the thresholds mentioned earlier in the research literature.

### 4.5. Comparison of Ni Concentrations with Literature Values

The release of Ni into the environment, a consequence of various industrial processes such as electroplating, heat treatment, use in electronic equipment, Ni-Cd battery industries, and tanneries [80], is not only a health concern but also a significant environmental issue. While Ni is an essential element naturally found in tissues [2], high concentrations of Ni can be toxic. Upon absorption, Ni accumulates in organs, including the brain, liver, kidney, bones, heart, and endocrine glands. Additionally, it can be deposited in nails, hair, and saliva [81]. Experimental research suggests that only 1–10% of dietary Ni^2+^ intake is absorbed from the gastrointestinal tract [28]. Once absorbed, Ni^2+^ is distributed throughout the body and accumulates primarily in the kidneys but is excreted mainly through feces and urine [28]. Ni can accumulate in the kidneys and testes, causing toxicity, and prolonged exposure can lead to cancer [2]. Researchers reported that Ni^2+^ can cause disorders and diseases at high concentrations, including cancer [2]. Ni^2+^ is known to have toxic effects on living organisms and is often considered a contaminant and a weak carcinogen [28]. Studies have shown that Ni^2+^ ingested by birds affects their respiratory system, leads to asthma, and damages DNA [81]. In birds from uncontaminated sites, the concentration of Ni in most avian species ranges from 0.1 to 5 µg·g^−1^ d.w. [2,28]. It has been reported that Ni concentrations of >3 and >10 µg·g^−1^ d.w. are toxic in wild birds’ liver and kidney tissues, respectively [69].

In this study, out of all the analyzed tissues, the highest Ni levels were recorded in the bone samples from adult feral pigeons and rooks in Iasi City, with the former exhibiting a Ni concentration of 1.099 ± 0.463 µg·g^−1^ w.w. and the latter registering a Ni level of 1.490 ± 0.499 µg·g^−1^ w.w. On the other hand, the tibia samples of pigeons from an industrial area in Drenas, Kosovo, displayed significantly lower Ni levels (0.41 ± 2 µg·g^−1^ d.w.) [28] than those in Iasi City. Furthermore, the highest Ni concentration for pigeons was found in the femur samples from the same industrial area in Kosovo, with a Ni level of 3.48 ± 1.01 µg·mg^−1^ d.w. [2]. Rook bone samples in Iasi City showed a slightly lower mean Ni concentration than that observed in the bone of a hooded crow (*Corvus cornix*) specimen from Rostovskaya Oblast, Azov District, Russia (0.8 µg·g^−1^ w.w.) [75]. However, the highest recorded Ni concentration in corvid species was found in the femur samples from house crows (*Corvus splendens*) in Visakhapatnam, India, with a Ni level of 12.96 ± 4.59 mg·kg^−1^ d.w. [80], surpassing the established environmental threshold for Ni of 0.1–5 µg·g^−1^ d.w. [2,28]. Adult feral pigeons and rooks in Iasi City had similar amounts of Ni in their livers, with mean values of 0.071 ± 0.031 µg·g^−1^ w.w. and 0.071 ± 0.037 µg·g^−1^ w.w., respectively. Notably, pigeons’ liver Ni levels were considerably higher worldwide, ranging from 1.01 ± 0.13 µg·g^−1^ w.w. in urban areas of Riyadh, Saudi Arabia [11], to 139.97 ± 67.8 µg·mg^−1^ d.w. in industrial areas of Drenas, Kosovo [2], which exceeded the toxic level for wild birds’ liver Ni of >10 µg·g^−1^ d.w. [69]. Rooks in various Polish environments exhibited lower or slightly higher Ni levels than in Iasi City, with measurements ranging from 0.15 ± 0.10 mg·kg^−1^ d.w. [82] to 0.2713 ± 0.088 mg·kg^−1^ d.w. [83]. Strikingly, higher corvid Ni liver levels were observed in Polish adult specimens of common ravens (*Corvus corax*), 0.626 ± 0.325 mg·kg^−1^ d.w. [83]. However, worldwide hepatic Ni values of rooks and related species were mainly within the background levels stated in the scientific literature for most avian species (0.1–5 µg·g^−1^ d.w.) [2,28].

### 4.6. Comparison of Pb Concentrations with Literature Values

It is crucial to emphasize that Pb^2+^ is a highly noxious metal that lacks any known biological function [2,34]. Even at minimal concentrations, it is toxic to living organisms due to its ability to inhibit hemoglobin synthesis by suppressing the delta-aminolevulinic acid enzyme. Chronic intoxication may result from long-term exposure to low doses of Pb [2]. Additionally, Pb^2+^ toxicity incidents have been reported in avian species, where it has been found to increase disease susceptibility [27,34]. The accumulation of Pb in the liver, kidneys, and bones can lead to toxicity and potentially cause cancer with long-term exposure [2]. The highest Pb concentrations were found in bone tissues, indicating that bones are the primary site of Pb deposition [69]. Pb content is similar in both the liver and kidneys, but bones retain the highest concentrations of Pb, making them a significant indicator of the total body burden of Pb [24,50]. Researchers have also found that Pb^2+^ disrupts Ca^2+^ homeostasis and the physiological role of Ca^2+^ in bones. Pb^2+^ has a greater affinity for the protein binding of Ca in bone, potentially displacing Ca [67]. Histological examination of pigeons’ lungs has revealed the accumulation of Pb particles, impairing lung function, particularly in high-traffic areas [24]. Background levels of Pb in various bird species range between 2 and 15 µg·g^−1^ d.w. in bone, 1 to 10 µg·g^−1^ d.w. in the kidney, and 0.5 to 5 µg·g^−1^ d.w. in the liver [65]. Franson and Pain (2011) [56] suggest a threshold level of 2 µg·g^−1^ w.w. for Pb in bird kidneys and liver, while other researchers propose a more limited environmental exposure threshold (0.65 µg·g^−1^ w.w.) for hepatic Pb concentrations [25]. Bone Pb concentrations above 5 µg·g^−1^ d.w. indicate exposure to Pb^2+^, while liver Pb concentrations of 1.7 µg·g^−1^ d.w. are commonly used to diagnose Pb intoxication [67]. Pb concentrations exceeding 6 µg·g^−1^ d.w. in bird livers cause sublethal toxicity [84], with subclinical toxicity levels ranging from 6 to 20 µg·g^−1^ d.w. in the liver [48].

Worldwide analysis of the research data on Pb concentrations in various tissues from feral pigeons and related species (Table S5 in Supplementary Materials) revealed that Iasi City’s adult feral pigeon hepatic mean value for Pb, 0.097 ± 0.109 µg·g^−1^ w.w., was slightly higher than the one measured at 0.089 ± 0.043 µg·g^−1^ w.w. in the livers of Spanish pigeons collected between 2016 and 2018 in urban habitats [25]. These figures are within the limits of environmental exposure for hepatic Pb mentioned above. However, the Pb liver mean concentration in Iasi City’s pigeons was significantly lower than the hepatic Pb levels detected in captive pigeons in urban areas in Saudi Arabia—20.87 ± 3.67 mg·g^−1^ w.w. [45]—which exceeded the Pb subclinical toxicity levels in the liver previously stated in the literature [48,84].

Furthermore, the value for Iasi City’s adult feral pigeons’ renal Pb concentration, 0.138 ± 0.141 µg·g^−1^ w.w., was below the range of Pb background levels of 1 to 10 µg·g^−1^ d.w. in the kidney of experimental specimens of adult bird species, as suggested by Scheuhammer (1987) [65], and lower than the threshold of 2 µg·g^−1^ w.w. for Pb in bird kidneys, expressed by Franson and Pain (2011) [56]. A similar, but slightly lower, mean Pb concentration was measured in the kidneys of pigeons in rural areas in Morocco—0.12 ± 0.03 mg·kg^−1^ w.w. [43]. These values were remarkably lower than the kidney Pb levels of pigeons in Drenas’ industrial environment in Kosovo—77.17 ± 30.8 µg·mg^−1^ d.w. [2]—which surpassed the Pb limits in renal tissue expressed before [56,65].

Pb concentrations in the pulmonary tissue of Iasi City pigeons revealed a mean value of 0.043 ± 0.037 µg·g^−1^ w.w., which was similar to the Pb level in the lungs of 5-year-old homing pigeons in urban areas in Guangzhou, China—173 ± 14 ng·g^−1^ d.w. [32]. However, these figures were much lower than the Pb levels measured in captive pigeons in urban areas in Saudi Arabia—17.87 ± 2.97 mg·g^−1^ w.w. [45].

Pigeon cardiac tissue in Iasi City registered a Pb mean value of 0.049 ± 0.035 µg·g^−1^ w.w., which was higher than the ones measured in pigeons’ heart samples in a rural habitat—0.02 ± 0.01 mg·kg^−1^ w.w.—and a high-traffic urban environment—0.02 ± 0.005 mg·kg^−1^ w.w.—in Morocco [43]. However, said values in Iasi City were significantly lower than those in various areas in the United Kingdom [85] and Saudi Arabia [11,45].

Pb concentrations in the pectoral muscle tissue of Iasi City pigeons discovered a mean value of 0.043 ± 0.033 µg·g^−1^ w.w., which was higher than the mean muscle Pb levels found in pigeons in urban habitats in Croatia—0.0035 mg·kg^−1^ w.w. [26]. On the other hand, Iasi City’s Pb values for muscle samples were remarkably lower than those in the muscles of pigeons in Bangladesh [9], Spain [23], United Kingdom [85], Korea [37], Saudi Arabia [11], and India [35].

The Pb level detected in pigeons’ sternums in Iasi City at 1.843 ± 1.176 µg·g^−1^ w.w. was similar to the mean Pb concentration measured in the bone samples of pigeons in rural habitats in Duckjuk Island, Korea—1.8 ± 0.86 µg·g^−1^ w.w. [18]. These figures were below the Pb environmental exposure levels in bone, as Scheuhammer (1987) stated, between 2 and 15 µg·g^−1^ d.w. [65]. However, the tibiotarsus samples of pigeons in urban areas in London registered Pb concentrations at 669.2 ± 45.5 µg·g^−1^ d.w. [22], significantly above the earlier threshold values.

After reviewing similar studies on Pb concentrations in various tissues from rooks and related species on a global scale (see Table S3 in Supplementary Materials), it can be concluded that the mean hepatic Pb value in rooks in Iasi City, 0.104 ± 0.106 µg·g^−1^ w.w., was slightly higher than the hepatic Pb concentration found in the livers of hooded crows (*Corvus cornix*) from urban areas in Cuneo Plain, Italy, which was recorded at 0.09 ± 0.27 mg·kg^−1^ w.w. [50]. The hepatic Pb concentration levels of corvids worldwide were mainly below the lowest environmental exposure threshold (0.65 µg·g^−1^ w.w.) for hepatic Pb concentrations [25] and the suggested threshold level of 2 µg·g^−1^ w.w. for Pb in bird liver by Franson and Pain (2011) [56]. However, the mean Pb concentration value for Malaysian house crows (*Corvus splendens*) from Klang was notably higher at 9.85 ± 5.54 µg·g^−1^ d.w. [69] and classified as Pb toxicosis according to the threshold level of 1.7 µg·g^−1^ d.w., as stated by Orłowski and his research team (2012) [67]. Nonetheless, corvids’ highest liver Pb levels were recorded in house crows (*Corvus splendens*) from India, quantified at 8.53 ± 1.12 µg·g^−1^ w.w. [35].

In this research study, the renal Pb concentration of the rooks in Iasi City was measured at 0.092 ± 0.081 µg·g^−1^ w.w., showing a slightly higher level compared to the hooded crows (*Corvus cornix*) from urban areas in Cuneo Plain, Italy, which were reported at 0.07 ± 0.10 mg·kg^−1^ w.w. [50]. This concentration was half compared to the levels of Pb found in the kidneys of jungle crows (*Corvus macrorhynchus*) from Teuri Island, Hokkaido, Japan, measuring 0.88 ± 0.43 mg·kg^−1^ d.w. [86]. Significant kidney Pb concentrations were also observed in the renal tissue of Polish rook nestlings from various urban environments, averaging 5.1 mg·kg^−1^ d.w. [67]. These levels were within the background levels of Pb in various bird species, which range between 1 and 10 µg·g^−1^ d.w. in the kidney [65] or below the threshold level of 2 µg·g^−1^ w.w. for Pb in bird kidneys, as suggested by Franson and Pain (2011) [56]. Furthermore, house crows (*Corvus splendens*) in the city of Klang, Malaysia [69], and in India [35] had the highest levels of Cd in their kidneys among corvids, measuring 21.47 ± 11.48 µg·g^−1^ d.w. and 9.20 ± 1.26 µg·g^−1^ w.w., respectively.

In the study conducted in Iasi City, the average concentration of Pb found in the pulmonary tissue of adult rooks was 0.029 ± 0.020 µg·g^−1^ w.w. This value indicates a significantly lower Pb level than the mean lung Pb concentration of rook nestlings from various cities in Poland, which measured at 6.0 mg·kg^−1^ d.w. [67]. Additionally, research on house crows (*Corvus splendens*) in the urban environment of Klang, Malaysia [69], revealed the highest amount of Pb in their lungs, measuring 13.33 ± 11.23 µg·g^−1^ d.w. This finding demonstrates a considerable disparity compared to the average observed in Iasi City, emphasizing the variance in Pb concentration levels among urban environments.

The average amount of Pb in rook cardiac tissue in Iasi City was 0.047 ± 0.032 µg·g^−1^ w.w., which was significantly lower than the Pb levels found in the hearts of house crows (*Corvus splendens*)—15.29 ± 7.54 µg·g^−1^ d.w.—in the urban environment of Klang, Malaysia [69].

Polish rook nestlings from various cities in Poland and house crows (*Corvus splendens*) in the urban area of Klang, Malaysia, exhibited high levels of Pb in their muscle tissue at 6.2 mg·kg^−1^ d.w. [67] and 5.06 ± 3.64 µg·g^−1^ d.w. [69], respectively. However, the highest amount of Pb was detected in the muscles of house crows (*Corvus splendens*) in India, quantified at 4.33 ± 0.45 µg·g^−1^ w.w. [35]. These values exceed the mean Pb concentration of the pectoral muscle tissue of adult rooks in Iasi City, 0.040 ± 0.035 µg·g^−1^ w.w.

A Malaysian research study uncovered that adult and juvenile specimens of house crows (*Corvus splendens*) in the urban area of Klang showed the highest average Pb value in their bones (23.44 ± 7.06 µg·g^−1^ d.w.) [69]. This figure exceeds the background bone Pb levels in various bird species, which usually range between 2 and 15 µg·g^−1^ d.w. [65]. On the other hand, the mean Pb concentration level in adult rooks’ sternums in Iasi City was significantly lower at 2.052 ± 1.420 µg·g^−1^ w.w. These results were similar to the bone Pb levels detected in rook specimens in the urban and suburban areas of Rostovskaya Oblast, Azov District, Russia, where the mean was determined to be 2.24 µg·g^−1^ w.w. [75].

## 5. Conclusions

The present study’s findings are significant as they provide insights into trace metal accumulation in the tissues of pigeons and rooks, both in general and specifically within Iasi City, Romania. Our research findings have yielded significant insights despite being constrained by limitations such as a small sample size and heterogeneity. Furthermore, the current scientific literature lacks established threshold values for the concentrations of all six trace elements analyzed in the feral pigeon and rook samples. These impediments have complicated the interpretation of our results on trace metal pollution. Consequently, we compared our findings with documented values from similar samples of feral pigeons, rooks, and related species in diverse habitats and pollution gradients. While existing studies have provided specific data on Cd and Pb concentrations in various sample types collected from feral pigeons and rooks, additional studies are needed on Co, Cr, Cu, and Ni concentrations in these species. Based on our research findings, the mean values of trace elements detected in the feral pigeon and rook specimens collected from Iasi City were below the environmental exposure thresholds outlined in the current scientific literature. These results prompt us to conclude that these bird specimens were not exposed to high levels of trace metals during their lives. However, it is essential to note that previous studies have suggested the hypothesis that prolonged exposure to even small amounts of toxic substances can have lasting effects on organisms. Therefore, we cannot discount the possibility of effects on the bird specimens collected in Iasi City. Nevertheless, our results have demonstrated similarities with the values reported in other studies and have revealed a slight correlation between trace metal concentrations in tissues and in the PM_10_ fraction of atmospheric particles. This correlation highlights the potential use of urban avian species as bioindicators for trace metal accumulation in urban environments. To enhance future research, we recommend increasing the sample size and monitoring the site fidelity of sampled birds. Furthermore, we propose including a reference site with updated environmental data and samples from a captive population to improve the results and their interpretation, particularly for trace metals with limited information. Consequently, we recommend further research on this topic using the histological analysis of tissues to uncover any potential long-term effects of trace metals on bird tissues from urban environments and point out links between trace element pollution through inhalation or ingestion.

## Figures and Tables

**Table 1:** Trace metal concentrations in the different organ, muscle, and bone samples of feral pigeons *Columba livia* f. *domestica* (*n* = 9) in Iasi City, which are expressed as the mean ± standard deviation (SD) on a µg·g^−1^ w.w. basis.

Trace Metal	Sample Type					
	Liver	Kidney	Lung	Heart	Muscle	Bone
Cd	0.094 ± 0.066	**0.190** ± 0.136	0.009 ± 0.007	0.008 ± 0.006	0.006 ± 0.006	0.015 ± 0.010
Co	0.006 ± 0.004	**0.009** ± 0.006	0.006 ± 0.003	0.007 ± 0.002	0.007 ± 0.002	0.008 ± 0.004
Cr	0.015 ± 0.017	0.017 ± 0.019	0.018 ± 0.021	0.018 ± 0.018	0.021 ± 0.018	**0.083** ± 0.038
Cu	**1.804** ± 0.663	1.711 ± 0.899	0.266 ± 0.065	1.674 ± 0.243	1.486 ± 0.421	0.174 ± 0.074
Ni	0.071 ± 0.031	0.073 ± 0.039	0.052 ± 0.039	0.056 ± 0.039	0.046 ± 0.044	**1.099** ± 0.463
Pb	0.097 ± 0.109	0.138 ± 0.141	0.043 ± 0.037	0.049 ± 0.035	0.043 ± 0.033	**1.843** ± 1.176

Note: The highest concentration values measured for each analyzed element are marked in bold.

**Table 2:** Trace metal concentrations in the different organ, muscle, and bone samples of rooks *Corvus frugilegus* (*n* = 6) in Iasi City, which are expressed as the mean ± SD on a µg·g^−1^ w.w. basis.

Trace Metal	Sample Type					
	Liver	Kidney	Lung	Heart	Muscle	Bone
Cd	0.169 ± 0.112	**0.356** ± 0.249	0.012 ± 0.008	0.013 ± 0.006	0.007 ± 0.006	0.014 ± 0.011
Co	0.009 ± 0.004	0.007 ± 0.006	0.009 ± 0.002	0.006 ± 0.005	0.009 ± 0.002	**0.011** ± 0.002
Cr	0.021 ± 0.027	0.032 ± 0.045	0.019 ± 0.018	0.025 ± 0.024	**0.146** ± 0.320	0.142 ± 0.055
Cu	1.666 ± 0.447	1.206 ± 0.399	0.275 ± 0.093	**1.910** ± 0.239	1.671 ± 0.188	0.203 ± 0.066
Ni	0.071 ± 0.037	0.060 ± 0.036	0.039 ± 0.032	0.039 ± 0.032	0.125 ± 0.202	**1.490** ± 0.499
Pb	0.104 ± 0.106	0.092 ± 0.081	0.029 ± 0.020	0.047 ± 0.032	0.040 ± 0.035	**2.052** ± 1.420

Note: The highest concentration values measured for each analyzed element are marked in bold.

**Table 3:** Trace metal annual mean concentrations in the PM_10_ fraction from atmospheric particles collected in Iasi City during 2016–2020 and the environmental threshold levels for reference [53].

Trace Metal	2016	2017	2018	2019	2020	Threshold	Unit of Measure
Cd	1.108	1.042	0.712	0.440	0.269	5.000	µg·m^−3^
Pb	0.026	0.045	0.042	0.029	0.024	0.500	ng·m^−3^
Ni	4.376	3.912	3.456	3.090	2.157	20.000	ng·m^−3^

## Data Availability

Data are contained within the article or Supplementary Materials.

## Supplementary Materials

The following supporting information can be downloaded at https://www.mdpi.com/article/10.3390/toxics12080593/s1, Table S1: Geographical coordinates for sampling sites, sampling dates, age, sex, and registered morphological indices of feral pigeons and rooks collected during 2019–2021 in Iasi City, Table S2: Cd concentrations in various organs, muscle, and bone samples of feral pigeons and related species from similar studies worldwide (values are as mentioned in the literature), Table S3: Cd, Co, Cr, Cu, Ni, and Pb concentrations in various organs, muscle, and bone samples of rooks and related corvid species from similar studies worldwide (values are as mentioned in the literature), Table S4: Co, Cr, Cu, and Ni concentrations in various organs, muscle, and bone samples of feral pigeons and related species from similar studies worldwide (values are as mentioned in the literature), Table S5: Pb concentrations in various organs, muscle, and bone samples of feral pigeons and related species from similar studies worldwide (values are as mentioned in the literature).
