# Supplementary material for: Trace Metal Bioaccumulation in Feral Pigeons (Columba livia f. domestica) and Rooks (Corvus frugilegus) Residing in the Urban Environment of Iasi City, Romania
Source: Toxics. 2024 Aug 16;12(8):593. doi: 10.3390/toxics12080593 (PMC11359549; doi:10.3390/toxics12080593)
Supplement: Supplementary file 1 [file toxics-12-00593-s001.zip › toxics-3124178-supplementary.pdf]

toxics

MDPI

Article

Trace Metal Bioaccumulation in Feral Pigeons (*Columba livia* f. *domestica*) and Rooks (*Corvus frugilegus*) Residing in the Urban Environment of Iasi City, Romania

Diana Iacob [1], Emanuela Paduraru [1], Vicentiu-Robert Gabor [2], Carmen Gache [3], Iuliana Gabriela Breaban [1,2], Silviu Gurlui [4], Gabriel Plavan [3], Roxana Jijie [5] and Mircea Nicoara [1,3,*]

1 Doctoral School of Geosciences, Faculty of Geography and Geology, Alexandru Ioan Cuza University of Iasi, No 20A Carol I Avenue, Iasi, 700505, Romania; dianaelenaiacob84@gmail.com (D.I.); emanuelapaduraru19@yahoo.com (E.P.)
2 Department of Geography, Faculty of Geography and Geology, Alexandru Ioan Cuza University of Iasi, No 20A Carol I Avenue, Iasi, 700505, Romania; gaborvicentiu@gmail.com (V.-R.G.); iulianab2001@yahoo.com (I.G.B.)
3 Department of Biology, Faculty of Biology, Alexandru Ioan Cuza University of Iasi, No 20A Carol I Avenue, Iasi, 700505, Romania; cgache@uaic.ro (C.G.); gabriel.plavan@uaic.ro (G.P.)
4 Faculty of Physics, Alexandru Ioan Cuza University of Iasi, No 11 Carol I Avenue, Iasi, 700506, Romania; sgurlui@uaic.ro (S.G.)
5 Research Center on Advanced Materials and Technologies (RAMTECH), Department of Exact and Natural Sciences, Institute of Interdisciplinary Research, Alexandru Ioan Cuza University of Iasi, No 11 Carol I Avenue, Iasi, 700506, Romania; roxana.jijie@uaic.ro (R.J.)
* Correspondence: mirmag@uaic.ro (M.N.).

Table S1. Geographical coordinates for sampling sites, sampling dates, age, sex, and registered morphological indices of feral pigeons and rooks collected during 2019–2021 in Iasi City.

Species	Sampling date	Age	Sex	Wing (cm)	Tail (cm)	Weight (g)	Sampling site	Latitude (° N)	Longitude (° E)
Columba livia f. *domestica*	11/28/2019	AD	M	23.0	12.5	180	IS1	47.174764	27.573640
Corvus frugilegus	10/20/2020	AD	F	30.8	18.0	353	IS1	47.174764	27.573640
Corvus frugilegus	3/3/2020	AD	M	31.0	19.1	489	IS2	47.171643	27.561322
Corvus frugilegus	7/18/2020	AD	F	30.0	17.0	373	IS2	47.171643	27.561322
Corvus frugilegus	11/16/2021	AD	M	31.0	18.5	470	IS2	47.171643	27.561322
Columba livia f. *domestica*	6/28/2020	AD	M	23.5	13.0	185	IS3	47.151797	27.571993
Columba livia f. *domestica*	9/23/2021	AD	M	23.2	11.5	189	IS3	47.151797	27.571993
Columba livia f. *domestica*	4/5/2021	AD	F	21.1	10.0	272	IS3	47.151797	27.571993
Columba livia f. *domestica*	11/23/2020	AD	M	23.3	13.0	200	IS4	47.174202	27.556781
Corvus frugilegus	1/21/2021	AD	M	30.0	16.0	385	IS4	47.174202	27.556781
Columba livia f. *domestica*	8/2/2021	AD	F	21.5	10.0	314	IS5	47.150884	27.586757
Columba livia f. *domestica*	8/2/2021	AD	M	23.5	12.0	223	IS5	47.150884	27.586757
Columba livia f. *domestica*	6/30/2021	AD	M	23.0	11.0	234	IS6	47.169670	27.577373
Columba livia f. *domestica*	5/19/2021	AD	M	23.5	12.5	207	IS7	47.174176	27.582114
Corvus frugilegus	7/8/2021	AD	M	27.0	14.0	281	IS8	47.155887	27.603800

Note: AD – adult specimens; M – male individuals; F – female individuals; IS1 to IS8 – sampling sites in Iasi (IS) City.

Table S2. Cd concentrations in various organs, muscle, and bone samples of feral pigeons and related species from similar studies worldwide (values are as mentioned in the literature).

Country	Study area	N	Sample type						Studied by
			Liver	Kidney	Lung	Heart	Muscle	Bone	
Bangladesh	Keranigonj, Norsinghdi, Mymensingh, Sirajgonj, Comilla [1]	60 [W,L]	1.1±0.086	0.93±0	NA	NA	0.03±0.006	NA	[9] [g,h,i]
Chile	Arica [1]	26 [W,L,F]	11.695±8.38	NA	NA	NA	NA	3.427±2.75	[31] [g,h,i]
China	Haidian District, Beijing [1]	15 [C,D,a]	382.8±59.3	2676±419	58.6±5.6	NA	NA	NA	[34] [j,h,k]
	Guangzhou [1]	10 [C,D,a]	649±60.3	7640.5±1411.1	112.1±8.2	NA	NA	NA	[33] [j,h,k]
		10 [C,D,b]	NA	NA	62±5	NA	NA	NA	[32] [j,h,k]
Croatia	Raša, Istria [1]	6 [W,L]	0.231	1.43	NA	NA	0.0045	NA	[26] [l,m,n]
India	Tamil Nadu, Gujarat, Kerala, Assam	24 [W,L]	8.59±1.53	13.19±3.09	NA	NA	1.89±1.06	NA	[35] [g,m,k]
Japan	Tokyo [8]	11 [W,L,c]	0.313±0.382	1.13±1.63	0.0489±0.057	NA	0.00874±0.00885	NA	[10] [g,h,i]
Korea	Seoul [3]	7 [W,L]	0.34±0.14	2.89±2.27	NA	NA	0.06±0.02	0.11±0.02	[37] [g,m,i]
	Seoul [4]	7 [W,L]	0.22±0.16	1.21±0.80	NA	NA	0.05±0.03	0.12±0.02	
	Seoul [6]	5 [W,L]	0.19±0.15	0.78±0.69	NA	NA	0.03±0.02	0.06±0.05	
	Seoul [7]	7 [W,L]	0.33±0.26	0.92±0.69	NA	NA	0.25±0.26	0.57±0.59	
	Hampyeong Park, Hampyeong [5]	6 [W,L]	0.881±0.211	4.463±1.406	0.086±0.007	NA	NA	ND	[38] [g,h,k]
	Hangang Park, Seoul [1]	6 [W,L]	1.45±0.238	8.773±4.23	0.083±0.008	NA	NA	ND	
	Kwangju [1]	9 [W,L]	0.08±0.02	0.18±0.06	NA	NA	NA	NA	[39] [g,m,i]
	Seoul [1]	12 [W,L]	0.24±0.08	1.05±0.62	NA	NA	NA	NA	
	Ansan [6]	10 [W,L]	0.14±0.06	0.43±0.28	0.21±0.13	NA	NA	0.27±0.08	[40] [g,m,i]
	Seoul [1]	12 [W,L]	0.24±0.08	1.05±0.62	0.22±0.04	NA	NA	0.23±0.07	
	Ansan [6]	10 [W,L]	0.14±0.05	0.43±0.27	0.21±0.13	NA	NA	NA	[18] [g,m,i]
	Busan [6]	9 [W,L]	0.25±0.12	0.66±0.72	0.19±0.05	NA	NA	NA	
	Duckjuk Island [5]	8 [W,L]	0.11±0.05	0.06±0.03	0.09±0.05	NA	NA	NA	
	Seoul [1]	12 [W,L]	0.24±0.08	1.05±0.62	0.22±0.03	NA	NA	NA	
Korea	Ulsan [6]	10 [W,L]	0.31±0.1	1.27±0.98	0.26±0.07	NA	NA	NA	[18] [g,m,i]
	Yochon [6]	11 [W,L]	0.21±0.05	0.68±0.58	0.2±0.04	NA	NA	NA	
Kosovo	Drenas [6]	2 [W,L]	0.85±0.2	19.1±3.6	NA	NA	NA	NA	[28] [g,h,i]
	Lubizhdë [5]	3 [W,L]	ND	10±11	NA	NA	NA	NA	
	Drenas [6]	20 [W,L,F,T]	0.52±0.34	0.88±0.61	NA	NA	NA	ND	[2] [j,h,i]
	Lubizhdë [5]	20 [W,L,F,T,d]	0.44±0.2	0.07±0.04	NA	NA	NA	ND	
Mexico	Ciudad de Mexico [1]	50 [W,L]	1.04	5.32	0.58	NA	NA	NA	[42] [l,h,n]
	Ixtlahuaca [5]	10 [W,L]	0.4	0.91	0.3	NA	NA	NA	

Country	Study area	N	Sample type						Studied by
			Liver	Kidney	Lung	Heart	Muscle	Bone	
Morocco	Allal Behraoui, Rabat-Salé 5	6 W,L	0.07±0.03	0.12±0.03	0.005±0.003	0.004±0.001	NA	NA	[43] l,m,i
	Kamra, Rabat-Salé 4	10 W,L	0.19±0.02	0.58±0.05	0.003±0.001	0.003±0.002	NA	NA	
	Oulja, Rabat-Salé 6	6 W,L	0.13±0.02	0.26±0.03	0.008±0.002	0.002±0.001	NA	NA	
	Rabat-Salé 3	9 W,L	0.2±0.04	3.07±1.11	0.03±0.007	0.019±0.008	NA	NA	
	Mimouza, Mohammedia 3	10 W,L	0.1±0.04	NA	NA	NA	NA	NA	[44] g,m,p
	Mohammedia 4	10 W,L	0.13±0.02	NA	NA	NA	NA	NA	
	Mohammedia 5	10 W,L	0.05±0.02	NA	NA	NA	NA	NA	
	Mohammedia 6	10 W,L	0.18±0.04	NA	NA	NA	NA	NA	
Netherlands	Amsterdam 3	8 W,L	0.53±0.5	2.51±2.8	0.03±0.01	NA	NA	NA	[21] g,m,i
	Amsterdam 4	8 W,L	0.43±0.29	2.73±2.61	0.02±0.01	NA	NA	NA	
	Assen 1	7 W,L	0.13±0.18	0.6±1.14	0.02±0.01	NA	NA	NA	
	Maastricht 1	5 W,L	0.27±0.3	0.67±0.83	0.02±0.01	NA	NA	NA	
Peru	Callao 6	9 W,L	0.247±0.283	NA	NA	NA	NA	NA	[41] l,m,i
	Lurín 5	6 W,L	0.054±0.078	NA	NA	NA	NA	NA	
	San Martín de Porres 1	6 W,L	0.19±0.102	NA	NA	NA	NA	NA	
Saudi Arabia	Riyadh 1	26 C,L,I	0.26±0.03	0.22±0.07	0.95±0.34	0.29±0.08	1.47±0.49	NA	[11] g,m,k
		14 W,D,I	0.11±0.001	NA	NA	NA	0.016±0.001	NA	[66] k,m,i
Saudi Arabia	Riyadh 1	34 W,D,N	0.046±0.001	NA	NA	NA	0.05±0.001	NA	[66] k,m,i
	Shaqraa Province, Riyadh 1	14 C,D,I	0.26±0.04	0.23±0.08	0.95±0.34	0.29±0.08	1.47±0.49	NA	[45] k,m,q
		4 C,D,N	10.65±1.42	8.55±0.28	7.98±0.93	7.87±0.87	3.76±0.67	NA	
Spain	Santa Cruz de Tenerife, Canary Islands 1	27 W,L,I	0.1096	0.6776	NA	NA	0.0075	NA	[23] g,m,n
United Kingdom	Cambridgeshire 5	5 W,L,e	0.54±0.05	1.75	NA	NA	NA	NA	[22] g,h,k
	Chelsea, London 1	43 W,L,f	2.45±0.28	12.3±2.05	NA	NA	NA	NA	
	Heathrow, Middlesex 8	15 W,L	9.48±3.15	50.7±22.7	NA	NA	NA	NA	
	Mortlake, London 7	15 W,L	0.4±0.07	1.52±0.31	NA	NA	NA	NA	

Note: N – sample number; 1 – urban; 2 – urban, light traffic; 3 – urban, medium traffic; 4 – urban, high traffic; 5 – rural; 6 – industrial; 7 – suburban; 8 – airport; W – wild specimens; C – captive specimens; I – specimens infected with parasites; N – specimens not infected with parasites; L – specimens of *Columba livia*; D – specimens of *Columba livia* f. *domestica*; U – specimens of *Columba livia* f. *urbana*; T – bone sample type is tibia; F - bone sample type is femur; NA – not assessed; ND – not detected; a – 5–6 yo homing pigeons specimens; b – 5 yo homing pigeons specimens; c – N=8 for

muscle samples; d – N=10 for kidney samples; e – N=2 for kidney samples; f – N=41 for kidney samples; g – values expressed as μg·g^{-1}; h – values expressed on a d.w. basis; i – values expressed as mean ± SD; j – values expressed as ng·g^{-1}; k – values expressed as mean ± SE; l – values expressed as mg·kg^{-1}; m – values expressed on a w.w. basis; n – values expressed as mean; o – values expressed as μg·mg^{-1}; p – values expressed as average ± SE; q – values expressed as mg·g^{-1}.

Table S3. Cd, Co, Cr, Cu, Ni, and Pb concentrations in various organs, muscle, and bone samples of rooks and related corvid species from similar studies worldwide (values are as mentioned in the literature).

Country	Study area	N	Sample type						Studied by
			Liver	Kidney	Lung	Heart	Muscle	Bone	
Cd									
India	Tamil Nadu, Gujarat, Kerala, Assam	19 W,S	8.78±1.87	10.52±5.25	NA	NA	5.544±3.56	NA	[35] h,i,j
Italy	Cuneo Plain (Caramagna Piemonte, Savigliano, Murello, Sant'Antonio Baligio, Racconigi, Moretta, Cavaller-maggiore) 1	124 W,Cn,a,b	0.15±0.26	0.39±0.95	NA	NA	NA	NA	[50] k,i,l
Japan	Teuri Island, Hokkaido	3 W,Ma	1.70±0.55	4.99±2.17	NA	NA	NA	NA	[86] k,m,l
	Tokyo 2	14 W,Ma,c	0.253±0.421	0.404±0.621	0.0360±0.0484	NA	0.00493±0.00236	NA	[10] h,m,l
		5 W,Co,d	0.353±0.277	0.599±0.526	0.105±0.115	NA	0.00554±0.00286	NA	
Malaysia	Klang 3	42 W,S,e	1.38±0.65	1.78±0.43	1.24±0.32	2.03±0.30	0.66±0.13	1.54±0.27	[69] h,m,l
Poland	Rzeszów, Białystok, Lublin, Warsaw 3	24 W,R	1.39±2.52	NA	NA	NA	NA	NA	[82] k,m,n
		6 W,Cn	0.62±0.17	NA	NA	NA	NA	NA	
		2 W,Mo	0.74	NA	NA	NA	NA	NA	[82] k,m,o
	Bezek Kolonia, Tarnogóra, Poturzyn, Podzamcze, Łysołaje 1	29 W,R,a	2.6±2.7	NA	NA	NA	NA	NA	[48] k,m,l
	Eastern and North Poland	9 W,R,a	2.511±1.421	NA	NA	NA	NA	NA	[83] k,m,l
		5 W,Ca,a	0.349±0.114	NA	NA	NA	NA	NA	
	Białystok, Lublin, and Rzeszów regions in Eastern Poland 1	7 W,R	1.98±0.72	NA	NA	NA	NA	NA	[49] k,m,l
	Ceranów, Podnieśno, Kotuń, Mokobody, Mordy, Stoczek, and Siedlce 1	35 W,R,F,f	17.2	17	17.2	NA	17.2	17.2	[67] k,m,p

Country	Study area	N	Sample type						Studied by
			Liver	Kidney	Lung	Heart	Muscle	Bone	
Co									
India	Visakhapatnam 3	11 W,S,F	ND	ND	NA	NA	ND	0.31±0.90	[80] h,m,l
Japan	Teuri Island, Hokkaido	3 W,Ma	0.15±0.09	0.25±0.09	NA	NA	NA	NA	[86] k,m,l
Poland	Eastern and North Poland	9 W,R,a	0.065±0.022	NA	NA	NA	NA	NA	[83] k,m,l
		5 W,Ca,a	0.033±0.004	NA	NA	NA	NA	NA	
	Ceranów, Podnieśno, Kotuń, Mokobody, Mordy, Stoczek, and Siedlce 1	35 W,R,F,f	4.2	4.1	4.1	NA	4.1	4.1	[67] k,m,p
Russia	Rostovskaya oblast, Azov District 3,4	2 W,R	NA	NA	NA	NA	NA	0.08	[75] h,i,q
		1 W,Cn	NA	NA	NA	NA	NA	0.09	
Cr									
India	Tamil Nadu, Gujarat, Kerala, Assam	19 W,S	15.85±4.65	18.10±6.71	NA	NA	10.83±2.96	NA	[35] h,i,j
Japan	Teuri Island, Hokkaido	3 W,Ma	0.11±0.03	0.06±0.01	NA	NA	NA	NA	[86] k,m,l
	Tokyo 2	14 W,Ma,c	0.422±0.136	0.588±0.153	0.815±0.363	NA	0.419±0.127	NA	[10] h,m,l
		5 W,Co	0.412±0.285	0.442±0.153	0.529±0.295	NA	0.364±0.206	NA	
Poland	Rzeszów, Białystok, Lublin, Warsaw 3	24 W,R	0.36±0.07	NA	NA	NA	NA	NA	[82] k,m,n
		6 W,Cn	0.45±0.15	NA	NA	NA	NA	NA	
	Rzeszów, Białystok, Lublin, Warsaw 3	2 W,Mo	0.3	NA	NA	NA	NA	NA	[82] k,m,o
	Bezek Kolonia, Tarnogóra, Poturzyn, Podzamcze, Łysołaje 1	29 W,R,a	0.4±0.1	NA	NA	NA	NA	NA	[48] k,m,l
	Eastern and North Poland	9 W,R,a	0.495±0.324	NA	NA	NA	NA	NA	[83] k,m,l
		5 W,Ca,a	0.507±0.347	NA	NA	NA	NA	NA	
	Białystok, Lublin, and Rzeszów regions in Eastern Poland 1	7 W,R	0.87±0.31	NA	NA	NA	NA	NA	[49] k,m,l
Russia	Rostovskaya oblast, Azov District 3,4	2 W,R	NA	NA	NA	NA	NA	0.59	[75] h,i,q
		1 W,Cn	NA	NA	NA	NA	NA	0.45	
	Rostov region 3	4 W,R,T,g	NA	NA	NA	NA	NA	5.63±3.7	[79] h,m,l
		6 W,R,T,a	NA	NA	NA	NA	NA	8.83±5.29	
	Rostov region 4	5 W,R,T,a	NA	NA	NA	NA	NA	5.0±6.1	

Country	Study area	N	Sample type Liver	Kidney	Lung	Heart	Muscle	Bone	Studied by
Cu									
India	Tamil Nadu, Gujarat, Kerala, Assam	19 W,S	45.03±10.64	38.96±23.96	NA	NA	42.28±11.52	NA	[35] h,i,j
	Visakhapatnam 3	11 W,S,F	ND	ND	NA	NA	ND	0.73±0.05	[80] h,m,l
Japan	Teuri Island, Hokkaido	3 W,Ma	20.7±7.14	15.4±1.31	NA	NA	NA	NA	[86] k,m,l
	Tokyo 2	14 W,Ma,c	42.5±15.8	14.9±1.76	4.76±1.3	NA	13.2±3.14	NA	[10] h,m,l
		5 W,Co	29.6±5.67	16.0±2.86	5.99±2.76	NA	13.3±3.31	NA	
Poland	Rzeszów, Białystok, Lublin, Warsaw 3	24 W,R	13.32±4.20	NA	NA	NA	NA	NA	[82] k,m,n
		6 W,Cn	16.61±5.93	NA	NA	NA	NA	NA	
		2 W,Mo	14.26	NA	NA	NA	NA	NA	[82] k,m,o
	Bezek Kolonia, Tarnogóra, Poturzyn, Podzamcze, Łysołaje 1	29 W,R,a	13.5±4.1	NA	NA	NA	NA	NA	[48] k,m,l
	Eastern and North Poland	9 W,R,a	11.64±3.1	NA	NA	NA	NA	NA	[83] k,m,l
		5 W,Ca,a	17.23±3.95	NA	NA	NA	NA	NA	
	Białystok, Lublin, and Rzeszów regions in Eastern Poland 1	7 W,R	11.80±2.46	NA	NA	NA	NA	NA	[49] k,m,l
	Ceranów, Podnieśno, Kotuń, Mokobody, Mordy, Stoczek, and Siedlce 1	35 W,R,F,f	3.1	2.1	2.1	NA	2.3	2.1	[67] k,m,p
Russia	Rostovskaya oblast, Azov District 3,4	2 W,R	NA	NA	NA	NA	NA	3.02	[75] h,i,q
		1 W,Cn	NA	NA	NA	NA	NA	4.16	
	Rostov region 3	4 W,R,T,g	NA	NA	NA	NA	NA	3.6±3.38	[79] h,m,l
		6 W,R,T,a	NA	NA	NA	NA	NA	4.77±2.5	
	Rostov region 4	5 W,R,T,a	NA	NA	NA	NA	NA	2.31±1.94	
Ni									
India	Visakhapatnam 3	11 W,S,F	ND	ND	NA	NA	0.08±0.99	12.96±4.59	[80] h,m,l
Japan	Teuri Island, Hokkaido	3 W,Ma	ND	ND	NA	NA	NA	NA	[86] k,m,l
Poland	Rzeszów, Białystok, Lublin, Warsaw 3	24 W,R	0.15±0.10	NA	NA	NA	NA	NA	[82] k,m,n
		6 W,Cn	0.09±0.13	NA	NA	NA	NA	NA	
		2 W,Mo	0.19	NA	NA	NA	NA	NA	[82] k,m,o

Country	Study area	N	Sample type						Studied by
			Liver	Kidney	Lung	Heart	Muscle	Bone	
Ni - continued									
Poland	Bezek Kolonia, Tarnogóra, Poturzyn, Podzamcze, Łysołaje $_{1}$	29 $_{W,R,a}$	0.2±0.1	NA	NA	NA	NA	NA	[48] $_{k,m,l}$
	Eastern and North Poland	9 $_{W,R,a}$	0.2713±0.088	NA	NA	NA	NA	NA	[83] $_{k,m,l}$
		5 $_{W,Ca,a}$	0.626±0.325	NA	NA	NA	NA	NA	
Russia	Rostovskaya oblast, Azov District $_{3,4}$	2 $_{W,R}$	NA	NA	NA	NA	NA	2.6	[75] $_{h,i,q}$
		1 $_{W,Cn}$	NA	NA	NA	NA	NA	0.8	
	Rostov region $_{3}$	4 $_{W,R,T,g}$	NA	NA	NA	NA	NA	1.75±0.45	[79] $_{h,m,l}$
		6 $_{W,R,T,a}$	NA	NA	NA	NA	NA	2.78±1.46	
	Rostov region $_{4}$	5 $_{W,R,T,a}$	NA	NA	NA	NA	NA	2.08±1.05	
Pb									
India	Tamil Nadu, Gujarat, Kerala, Assam	19 $_{W,S}$	8.53±1.12	9.20±1.26	NA	NA	4.33±0.45	NA	[35] $_{h,i,j}$
Italy	Cuneo Plain (Caramagna Piemonte, Savigliano, Murello, Sant'Antonio Baligio, Racconigi, Moretta, Cavaller-maggiore) $_{1}$	124 $_{W,Cn,a,b}$	0.09±0.27	0.07±0.10	NA	NA	NA	NA	[50] $_{k,i,l}$
Japan	Teuri Island, Hokkaido	3 $_{W,Ma}$	0.47±0.15	0.88±0.43	NA	NA	NA	NA	[86] $_{k,m,l}$
Malaysia	Klang $_{3}$	42 $_{W,S,e}$	9.85±5.54	21.47±11.48	13.33±11.23	15.29±7.54	5.06±3.64	23.44±7.06	[69] $_{h,m,l}$
Poland	Rzeszów, Białystok, Lublin, Warsaw $_{3}$	24 $_{W,R}$	1.12±1.42	NA	NA	NA	NA	NA	[82] $_{k,m,n}$
		6 $_{W,Cn}$	3.14±7.35	NA	NA	NA	NA	NA	
		2 $_{W,Mo}$	2.25	NA	NA	NA	NA	NA	[82] $_{k,m,o}$
	Bezek Kolonia, Tarnogóra, Poturzyn, Podzamcze, Łysołaje $_{1}$	29 $_{W,R,a}$	1.5±1.4	NA	NA	NA	NA	NA	[48] $_{k,m,l}$
	Eastern and North Poland	9 $_{W,R,a}$	2.11±1.865	NA	NA	NA	NA	NA	[83] $_{k,m,l}$
		5 $_{W,Ca,a}$	0.92±0.149	NA	NA	NA	NA	NA	
	Białystok, Lublin, and Rzeszów regions in Eastern Poland $_{1}$	7 $_{W,R}$	2.35±0.88	NA	NA	NA	NA	NA	[49] $_{k,m,l}$

Country	Study area	N	Sample type						Studied by
			Liver	Kidney	Lung	Heart	Muscle	Bone	
Pb - continued									
Poland	Ceranów, Podnieśno, Kotuń, Mokobody, Mordy, Stoczek, and Siedlce 1	35 W,R,F,f	5	5.1	6	NA	6.2	6	[67] k,m,p
Russia	Rostovskaya oblast, Azov District 3,4	2 W,R	NA	NA	NA	NA	NA	2.24	[75] h,i,q
		1 W,Cn	NA	NA	NA	NA	NA	0.92	
	Rostov region 3	4 W,R,T,g	NA	NA	NA	NA	NA	7.9±6.4	[79] h,m,l
		6 W,R,T,a	NA	NA	NA	NA	NA	8.69±6.15	
	Rostov region 4	5 W,R,T,a	NA	NA	NA	NA	NA	4.54±4.9	

Note: N – sample number; 1 – rural; 2 – airport; 3 – urban; 4 – suburban; W – wild specimens; S – *Corvus splendes*; Cn – *Corvus cornix*; Ma – *Corvus macrorhynchus*; Co – *Corvus corone*; R – *Corvus frugilegus*; Mo – *Corvus monedula*; Ca – *Corvus corax*; F – bone sample type is femur; T – bone sample type is tarsus; a – adult specimens; b – N=126 for kidney samples; c – N=12 for muscle samples; d – N=4 for muscle samples; e – adult and juvenile specimens; f – nestlings specimens; g – juvenile specimens; h – values expressed as $\mu g \cdot g^{-1}$; i – values expressed on a w.w. basis; j – values expressed as mean ± standard error; k – values expressed as $mg \cdot kg^{-1}$; l – values expressed as mean ± SD; m – values expressed on a d.w. basis; n – values expressed as geometric mean ± SD; o – values expressed as arithmetic mean; p – values expressed as average; q – values expressed as mean; NA – not assessed; ND – not detected.

Table S4. Co, Cr, Cu, and Ni concentrations in various organs, muscle, and bone samples of feral pigeons and related species from similar studies worldwide (values are as mentioned in the literature).

Country	Study area	N	Sample type						Studied by
			Liver	Kidney	Lung	Heart	Muscle	Bone	
Co									
Pakistan	Kasur, Punjab 5	5 W,L	0.03	1.56	4.54	NA	0.5	NA	[71] k,l,m
	Lahore, Punjab 3	5 W,L	0.29	0.56	2.75	NA	0.1	NA	
	Shahdara, Punjab 3,5	5 W,L	0.2	0.9	1.97	NA	1.55	NA	
Saudi Arabia	Riyadh 1	26 C,L,I	0.22±0.09	0.67±0.15	4.28±3.85	0.21±0.14	1.58±0.59	NA	[11] n,o,p
		14 W,D,I	0.104±0.01	NA	NA	NA	0.085±0.01	NA	[66] q,o,r
		34 W,D,N	0.212±0.01	NA	NA	NA	0.473±0.01	NA	
	Shaqraa Province, Riyadh 1	4 C,D,N	20.76±2.12	32.9±4.98	56.32±6.98	19.76±2.97	45.98±2.76	NA	[45] q,o,p
		14 C,D,I	0.23±0.09	0.67±0.05	4.28±3.85	0.21±0.04	1.58±0.59	NA	[45] q,o,p
Cr									
Bangladesh	Keranigonj, Norsinghdi, Mymensingh, Sirajgonj, Comilla 1	60 W,L	3.37±0	2.74±0.086	NA	NA	0.91±0.086	NA	[9] n,l,r

Country	Study area	N	Sample type						Studied by
			Liver	Kidney	Lung	Heart	Muscle	Bone	
Cr - continued									
India	Tamil Nadu, Gujarat, Kerala, Assam	24 W,L	8.59±1.53	31.92±16.64	NA	NA	3.49±1.08	NA	[35] n,o,p
Japan	Tokyo 7	11 W,L	0.207±0.0502	0.279±0.172	0.385±0.149	NA	0.196±0.0623	NA	[10] n,l,r
Mexico	Ciudad de Mexico 1	50 W,L	1.77	4.34	2.66	NA	NA	NA	[42] k,l,m
	Ixtlahuaca 4	10 W,L	0.62	1.17	0.85	NA	NA	NA	
Pakistan	Kasur, Punjab 5	5 W,L	2.83	6.25	9.28	NA	8.18	NA	[71] k,l,m
	Lahore, Punjab 3	5 W,L	2.59	6.7	5.05	NA	6.54	NA	
	Shahdara, Punjab 3,5	5 W,L	3.33	7	11.2	NA	12.8	NA	
Saudi Arabia	Riyadh 1	26 C,L,I	1.37±1.13	1.69±0.49	0.51±0.08	0.32±0.73	0.53±0.19	NA	[11] n,o,p
		14 W,D,I	0.341±0.01	NA	NA	NA	0.092±0.001	NA	[66] q,o,r
		34 W,D,N	0.617±0.02	NA	NA	NA	0.592±0.02	NA	
	Shaqraa Province, Riyadh 1	4 C,D,N	16.76±3.54	9.76±0.87	5.76±0.78	8.76±0.96	5.04±0.89	NA	[45] q,o,p
		14 C,D,I	1.37±0.03	1.69±0.04	0.51±0.08	0.32±0.07	0.53±0.19	NA	
Spain	Santa Cruz de Tenerife, Canary Islands 1	27 W,L,I	0.5206	0.5134	NA	NA	0.6094	NA	[23] n,o,m
Cu									
Bangladesh	Keranigonj, Norsinghdi, Mymensingh, Sirajgonj, Comilla 1	60 W,L	35.02±0.135	12.33±0	NA	NA	5.48±0	NA	[9] n,l,r
Croatia	Raša, Istria 1	6 W,L	3.56	3.87	NA	NA	5.12	NA	[26] k,o,m
India	Tamil Nadu, Gujarat, Kerala, Assam	24 W,L	38.75±3.80	17.72±2.88	NA	NA	42.54±4.48	NA	[35] n,o,p
Iraq	Al-Nassiriyah 1	129 C,L,I,a	0.799±0.07	0.61±0.16	NA	NA	NA	NA	[1] n,l,r
		171 C,L,N,b	0.164±0.02	0.078±0.017	NA	NA	NA	NA	
Japan	Tokyo 7	11 W,L	48.6±46.7	13.1±3.44	3.58±1.18	NA	15.2±2.14	NA	[10] n,l,r
Korea	Seoul 2	7 W,L	10.86±24.89	134.25±152.97	NA	NA	14.95±20.51	36.11±62.08	[37] n,o,r
	Seoul 3	7 W,L	1.38±0.44	1.30±0.08	NA	NA	2.51±0.66	1.22±0.46	
	Seoul 5	5 W,L	1.61±0.80	1.42±0.75	NA	NA	2.71±0.72	1.34±0.82	
	Seoul 6	7 W,L	3.49±2.67	2.28±1.49	NA	NA	3.15±0.40	2.63±2.30	
Kosovo	Drenas 5	10 W,L	10.5±5.3	9.8±8.9	NA	NA	NA	NA	[28] n,l,r
	Lubizhdë 4	10 W,L,c	8±4	18±23	NA	NA	NA	NA	
	Koshare 4	10 W,T	NA	NA	NA	NA	NA	2.9±2.9	[29] n,l,r
	Mitrovica 5	10 W,D,T	NA	NA	NA	NA	NA	2.3±0.7	
	Mitrovica 5	10 W,U,T	NA	NA	NA	NA	NA	1.8±0.6	

Country	Study area	N	Sample type Liver	Kidney	Lung	Heart	Muscle	Bone	Studied by
Cu - continued									
Kosovo	Drenas 5	20 W,L,F,d	18.48±5.43	29.16±8.08	NA	NA	NA	0.8±0.3	[2] s,l,r
		20 W,L,T	NA	NA	NA	NA	NA	2.74±1.05	
	Lubizhdë 4	8 W,L,T	NA	NA	NA	NA	NA	1.12±0.44	
		20 W,L,F,e	13.08±3.38	12.58±5.76	NA	NA	NA	4.27±1.76	
Pakistan	Kasur, Punjab 5	5 W,L	0.25	0.56	0.68	NA	0.73	NA	[71] k,l,m
	Lahore, Punjab 3	5 W,L	0.32	0.56	0.25	NA	0.86	NA	
	Shahdara, Punjab 3,5	5 W,L	0.3	0.48	0.49	NA	0.68	NA	
Peru	Callao 5	9 W,L	6.744±3.367	NA	NA	NA	NA	NA	[41] k,o,r
	Lurín 4	6 W,L	3.135±1.928	NA	NA	NA	NA	NA	
	San Martín de Porres 1	6 W,L	3.5±0.785	NA	NA	NA	NA	NA	
Saudi Arabia	Riyadh 1	26 C,L,I	12.7±1.2	9.44±0.8	4.62±0.55	4.62±0.59	13.01±2.1	NA	[11] n,o,p
		14 W,D,I	0.132±0.01	NA	NA	NA	0.128±0.01	NA	[66] q,o,r
		34 W,D,N	0.608±0.02	NA	NA	NA	0.582±0.02	NA	
	Shaqraa Province, Riyadh 1	4 C,D,N	30.65±4.56	27.87±3.77	23.77±3.66	16.87±2.88	35.87±4.66	NA	[45] q,o,p
		14 C,D,I	12.65±1.21	9.45±0.84	4.85±0.39	4.62±0.59	13.02±2.06	NA	
Spain	Santa Cruz de Tenerife, Canary Islands 1	27 W,L,I	3.407	2.841	NA	NA	4.023	NA	[23] n,o,m
Ni									
Kosovo	Drenas 5	10 W,L,T,f	7.4±15.5	21.4±38	NA	NA	NA	0.41±2	[28] n,l,r
		10 W,L,F	NA	NA	NA	NA	NA	9.3±10	
	Lubizhdë 4	10 W,L,T,F	ND	ND	NA	NA	NA	ND	
	Drenas 5	20 W,L,F,g	139.97±67.8	18.57±8.76	NA	NA	NA	3.48±1.01	[2] s,l,r
		12 W,L,T,h	NA	NA	NA	NA	NA	3.42±1.48	
	Lubizhdë 4	10 W,L,F,i	2.75±1.03	8.33±3.47	NA	NA	NA	1.35±0.22	
		2 W,L,T,j	NA	NA	NA	NA	NA	0.82±0.19	
Saudi Arabia	Riyadh 1	26 C,L,I	1.01±0.13	3.47±0.71	1.31±0.12	0.77±0.08	2.79±0.22	NA	[11] n,o,p
	Shaqraa Province, Riyadh 1	4 C,D,N	30.85±7.74	40.75±9.74	60.76±9.57	25.87±10.76	63.87±8.67	NA	[45] q,o,p
		14 C,D,I	1.02±0.13	3.47±0.71	1.31±0.12	0.77±0.08	2.79±0.22	NA	

Note: N – sample number; 1 – urban; 2 – urban, medium traffic; 3 – urban, high traffic; 4 – rural; 5 – industrial; 6 – suburban; 7 – airport; W – wild specimens; C – captive specimens; I – specimens infected with parasites; N – specimens not infected with parasites; L – specimens of *Columba livia*; D – specimens of *Columba livia* f. *domestica*; U – specimens of *Columba livia* f. *urbana*; T – bone sample

type is tibia; F - bone sample type is femur; NA – not assessed; ND – not detected; a – liver results for summer and kidney results for spring; b – liver results for summer and kidney results for winter; c – N=2 for kidney samples; d – N=12 for bone samples; e – N=9 for bone samples; f – N=7 for liver samples; g – N=14 for liver samples and N=12 for bone samples; h – N=12 for bone samples; i – N=2 for bone samples; j – N=2 for bone samples; k – values expressed as mg·kg^{-1}; l – values expressed on a d.w. basis; m – values expressed as mean; n – values expressed as μg·g^{-1}; o – values expressed on a w.w. basis; p – values expressed as mean ± SE; q – values expressed as mg·g^{-1}; r – values expressed as mean ± SD; s – values expressed as μg·mg^{-1}.

Table S5. Pb concentrations in various organs, muscle, and bone samples of feral pigeons and related species from similar studies worldwide (values are as mentioned in the literature).

Country	Study area	N	Sample type						Studied by
			Liver	Kidney	Lung	Heart	Muscle	Bone	
Bangladesh	Keranigonj, Norsinghdi, Mymensingh, Sirajgonj, Comilla $_{1}$	60 $_{W,L}$	2.85±0.01	1.17±0.006	NA	NA	0.39±0.029	NA	[9] $_{n,o,p}$
Chile	Arica $_{1}$	26 $_{W,L,F}$	1.702±1.26	NA	NA	NA	NA	10.845±6.03	[31] $_{n,o,p}$
China	Haidian District, Beijing $_{1}$	15 $_{C,D,a}$	200.2±28.2	441±37.1	261±22.2	NA	NA	NA	[34] $_{q,o,r}$
	Guangzhou $_{1}$	10 $_{C,D,a}$	240.3±43.1	364.4±35.2	442.1±23.5	NA	NA	NA	[33] $_{q,o,r}$
		10 $_{C,D,b}$	NA	NA	173±14	NA	NA	NA	[32] $_{q,o,r}$
Croatia	Raša, Istria $_{1}$	6 $_{W,L}$	0.0238	0.0728	NA	NA	0.0035	NA	[26] $_{s,t,u}$
Germany	Munich $_{2}$	50 $_{W,U,F}$	NA	NA	NA	NA	NA	10.69±3.59	[30] $_{s,t,v}$
	Munich $_{3}$	50 $_{W,U,F}$	NA	NA	NA	NA	NA	27.88±5.3	
	Munich $_{4}$	50 $_{W,U,F}$	NA	NA	NA	NA	NA	49.47±6.93	
	Upper Bavaria $_{5}$	50 $_{C,D,F}$	NA	NA	NA	NA	NA	1.04±0.95	
India	Tamil Nadu, Gujarat, Kerala, Assam	24 $_{W,L}$	6.45±0.82	14.48±8.56	NA	NA	3.48±0.58	NA	[35] $_{n,t,r}$
Iraq	Al-Nassiriyah $_{1}$	129 $_{C,L,I,c}$	0.589±0.09	0.44±0.14	NA	NA	NA	NA	[1] $_{n,o,p}$
		171 $_{C,L,N,d}$	0.071±0.01	0.056±0.014	NA	NA	NA	NA	
Korea	Kyongju $_{2}$	13 $_{W,L}$	1.42±0.31	1.21±0.31	1.04±0.19	NA	NA	5.78±0.86	[36] $_{n,t,r}$
	Taegu $_{1}$	20 $_{W,L}$	1.82±0.38	2.4±0.52	0.85±0.12	NA	NA	6.22±0.82	
	Taegu $_{4}$	14 $_{W,L}$	4.42±1.16	6.07±1.61	1.85±0.23	NA	NA	14.87±1.28	
	Seoul $_{3}$	7 $_{W,L}$	1.66±0.87	5.61±3.36	NA	NA	1.47±0.67	21.67±14.08	[37] $_{n,t,p}$
	Seoul $_{4}$	7 $_{W,L}$	1.45±0.89	6.13±2.43	NA	NA	0.73±0.35	52.07±21.93	
	Seoul $_{6}$	5 $_{W,L}$	1.38±0.18	6.44±5.85	NA	NA	0.73±0.18	16.75±12.73	
	Seoul $_{7}$	7 $_{W,L}$	1.13±0.75	5.75±6.00	NA	NA	3.33±6.1	7.77±3.70	
	Hampyeong Park, Hampyeong $_{5}$	6 $_{W,L}$	0.425±0.057	1.490±0.182	0.376±0.039	NA	NA	4.858±1.232	[38] $_{n,o,r}$
	Hangang Park, Seoul $_{1}$	6 $_{W,L}$	0.968±0.158	2.116±0.393	0.488±0.104	NA	NA	16.621±5.769	[38] $_{n,o,r}$
	Kwangju $_{1}$	9 $_{W,L}$	1.64±0.99	NA	NA	NA	NA	24.4±19.7	[39] $_{n,t,p}$
	Seoul $_{1}$	12 $_{W,L}$	2.33±0.78	NA	NA	NA	NA	29.5±21.1	

Country	Study area	N	Sample type						Studied by
			Liver	Kidney	Lung	Heart	Muscle	Bone	
Korea	Ansan 6	10 W,L	1.8±0.46	2.98±1.38	1.58±0.37	NA	NA	10.5±4.69	[40] n,t,p
	Seoul 1	12 W,L	2.33±0.78	4.13±1.131	1.72±0.66	NA	NA	29.5±21.1	
	Ansan 6	12 W,L	1.87±0.52	NA	NA	NA	NA	NA	[8] n,t,p
	Busan 6	11 W,L	2.02±0.63	NA	NA	NA	NA	NA	
	Duckjeok 5	8 W,L	1.57±0.27	NA	NA	NA	NA	NA	
	Seoul 1	10 W,L	2.09±0.57	NA	NA	NA	NA	NA	
	Ulsan 6	9 W,L	1.68±0.48	NA	NA	NA	NA	NA	
	Yochon 6	17 W,L	1.44±0.38	NA	NA	NA	NA	NA	
	Ansan 6	10 W,L	1.8±0.46	2.98±1.38	1.58±0.37	NA	NA	10.5±4.7	[18] n,t,p
	Busan 6	9 W,L	2.72±1.49	4.03±2.41	1.94±0.52	NA	NA	23.8±13.7	
	Duckjuk Island 5	8 W,L	1.57±0.27	1.45±0.24	0.94±0.4	NA	NA	1.8±0.86	
	Seoul 1	12 W,L	2.33±0.78	4.13±1.31	1.71±0.66	NA	NA	29.5±21.1	
	Ulsan 6	10 W,L	1.84±1.2	3.29±3.3	1.8±0.67	NA	NA	24.6±21.4	
	Yochon 6	11 W,L	1.36±0.27	2.52±0.75	1.55±0.32	NA	NA	2.13±0.8	
Kosovo	Drenas 6	10 W,L,T	NA	NA	NA	NA	NA	35.9±34	[28] n,o,p
		10 W,L,F	NA	NA	NA	NA	NA	51.4±43	
	Lubizhdë 5	10 W,L,T,F	NA	NA	NA	NA	NA	ND	
	Koshare 5	10 W,L,T	NA	NA	NA	NA	NA	7.7±8.1	[29] n,o,p
	Mitrovica 6	10 W,D,T	NA	NA	NA	NA	NA	188±101	
		10 W,U,T	NA	NA	NA	NA	NA	530±583	
		20 W,L,F	NA	NA	NA	NA	NA	151±110	[16] n,o,p
	Drenas 6	20 W,L,F	17.43±8.13	77.17±30.8	NA	NA	NA	58.38±22.93	[2] w,o,p
		20 W,L,T	NA	NA	NA	NA	NA	44.93±16.69	
	Lubizhdë 5	12 W,L,F,e	13.04±5.95	60.69±25.5	NA	NA	NA	4.27±1.76	
		4 W,L,T	NA	NA	NA	NA	NA	6.02±0.94	
Mexico	Ciudad de Mexico 1	50 W,L	3.93	7.61	3.65	NA	NA	NA	[42] s,o,u
	Ixtlahuaca 5	10 W,L	1.09	1.92	1.17	NA	NA	NA	
Morocco	Allal Behraoui, Rabat-Salé 5	6 W,L	0.07±0.01	0.12±0.03	0.05±0.02	0.02±0.01	NA	NA	[43] s,t,p
	Kamra, Rabat-Salé 4	10 W,L	0.12±0.01	0.29±0.02	0.1±0.01	0.02±0.005	NA	NA	
	Oulja, Rabat-Salé 6	6 W,L	0.56±0.05	0.56±0.06	0.12±0.03	0.11±0.03	NA	NA	
	Rabat-Salé 3	9 W,L	0.37±0.06	0.48±0.04	0.07±0.02	0.06±0.01	NA	NA	
	Mimouza, Mohammedia 3	10 W,L	0.17±0.06	NA	NA	NA	NA	NA	[44] n,t,x
	Mohammedia 4	10 W,L	0.39±0.16	NA	NA	NA	NA	NA	
	Mohammedia 5	10 W,L	0.05±0.02	NA	NA	NA	NA	NA	

Country	Study area	N	Sample type						Studied by
			Liver	Kidney	Lung	Heart	Muscle	Bone	
Morocco	Mohammedia 6	10 W,L	0.82±0.27	NA	NA	NA	NA	NA	[44] n,t,x
Netherlands	Amsterdam 3	8 W,L	0.18±0.06	0.53±0.15	0.47±0.27	NA	NA	NA	[21] n,t,p
	Amsterdam 4	8 W,L	1.21±0.75	2.44±1.30	0.65±0.37	NA	NA	NA	
	Assen 1	7 W,L	0.16±0.09	0.41±0.18	0.25±0.12	NA	NA	NA	
	Maastricht 1	5 W,L	0.13±0.12	0.30±0.19	0.31±0.13	NA	NA	NA	
Peru	Callao 6	9 W,L	0.624±0.701	NA	NA	NA	NA	NA	[41] s,t,p
	Lurín 5	6 W,L	0.665±0.564	NA	NA	NA	NA	NA	
	San Martín de Porres 1	6 W,L	0.073±0.075	NA	NA	NA	NA	NA	
Saudi Arabia	Riyadh 1	26 C,L,I	1.79±0.82	1.14±0.21	0.86±0.13	1.04±0.14	1.55±0.51	NA	[11] n,t,r
	Shaqraa Province, Riyadh 1	4 C,D,N	20.87±3.67	19.76±2.76	17.87±2.97	23.77±3.96	30.76±3.65	NA	[45] y,t,r
		14 C,D,I	1.79±0.63	1.89±0.83	0.86±0.13	1.12±0.13	1.72±0.40	NA	
Slovakia	Bratislava 6	22 W,L,TM,f	NA	NA	NA	NA	NA	29.7±6.4	[27] n,o,r
		22 W,L,TM,g	NA	NA	NA	NA	NA	50.8±12.6	
	Bratislava 4,6	39 W,L,T	NA	NA	NA	NA	NA	2.65±0.50	[20] s,o,r
Spain	Madrid 2	25 W,L,i	NA	NA	7.04±1.85	NA	NA	75.75±37.89	[24] n,o,p
	Madrid 3	15 W,L,j	NA	NA	7.71±0.91	NA	NA	148.19±37.63	
	Madrid 4	36 W,L,k	NA	NA	11.21±2.32	NA	NA	144.19±44.01	
	Madrid 5	6 W,L,h	NA	NA	0.58±0.2	NA	NA	15.04±1.09	
	Alicante, Comunidad Valenciana 5	30 W,L	0.02±0.008	NA	NA	NA	NA	NA	[25] n,t,z
	La Gomera, Santa Cruz de Tenerife, Canary Islands 5	1 W,L	0.165	NA	NA	NA	NA	NA	
	Madrid 7	10 W,L	0.187±0.103	NA	NA	NA	NA	NA	
	Valencia, Comunidad Valenciana 1	30 W,L	0.089±0.043	NA	NA	NA	NA	NA	
	Zamora, Castilla-Léon 5	28 W,L	0.022±0.01	NA	NA	NA	NA	NA	
	Santa Cruz de Tenerife, Canary Islands 1	27 W,L,I	0.2907	0.287	NA	NA	0.1108	NA	[23] n,t,u
United Kingdom	Cambridgeshire 5	7 W,L,TT,l	2.01±0.29	4.34±1.26	NA	NA	NA	5.73±1.05	[22] n,o,r
	Chelsea, London 1	53 W,L,TT,m	21.6±1.95	321.4±45.3	NA	NA	NA	669.2±45.5	
	Heathrow, Middlesex 8	15 W,L,TT	6.11±1.09	9.87±2.6	NA	NA	NA	107.9±27.5	
	Mortlake, London 7	15 W,L,TT	10.1±2.36	48.7±17.5	NA	NA	NA	281.8±73.7	

Country	Study area	N	Sample type						Studied by
			Liver	Kidney	Lung	Heart	Muscle	Bone	
United Kingdom	Bridport, Dorset 5	8 W,L,TT	2.3±0.6	6.3±2.0	NA	1.8±0.7	1.8±0.2	12±1.9	[85] n,o,r
	Dorchester, Dorset 7	8 W,L,TT	6.5±1.7	17±4.4	NA	2.3±0.6	2.2±0.3	33±6	
	Liverpool 1	12 W,L,TT	13.7±1.6	189±52	NA	4.7±0.6	3.7±0.6	245±41	

Note: N – sample number; 1 – urban; 2 – urban, light traffic; 3 – urban, medium traffic; 4 – urban, high traffic; 5 – rural; 6 – industrial; 7 – suburban; 8 – airport; W – wild specimens; C – captive specimens; I – specimens infected with parasites; N – specimens not infected with parasites; L – specimens of *Columba livia*; D – specimens of *Columba livia* f. *domestica*; U – specimens of *Columba livia* f. *urbana*; T – bone sample type is tibia; F - bone sample type is femur; TM - bone sample type is tarsometatarsi; TT - bone sample type is tibiotarsus; NA – not assessed; ND – not detected; a – 5–6 yo homing pigeons specimens; b – 5 yo homing pigeons specimens; c – liver results for summer and kidney results for spring; d – liver results for summer and kidney results for autumn; e – N=10 for bone samples and N=13 for kidney samples; f – wild type specimens; g – urban type specimens; h – N=3 for bone samples and results from bone samples only for female specimens; i – N=12 for bone samples and results from bone samples only for female specimens; j – N=6 for bone samples and results from bone samples only for female specimens; k – N=20 for bone samples and results from bone samples only for female specimens; l – N=10 for liver samples; m – N=41 for bone samples and N=64 for kidney samples; n – values expressed as $\mu g \cdot g^{-1}$; o – values expressed on a d.w. basis; p – values expressed as mean ± SD; q – values expressed as $ng \cdot g^{-1}$; r – values expressed as mean ± SE; s – values expressed as $mg \cdot kg^{-1}$; t – values expressed on a w.w. basis; u – values expressed as mean; v – values expressed as arithmetic mean ± SD; w – values expressed as $\mu g \cdot mg^{-1}$; x – values expressed as average ± SE; y – values expressed as $mg \cdot g^{-1}$; z – values expressed as geometric mean ± SE.
